# Extended Evaluation of Virological, Immunological and Pharmacokinetic Endpoints of CELADEN: A Randomized, Placebo-Controlled Trial of Celgosivir in Dengue Fever Patients

**DOI:** 10.1371/journal.pntd.0004851

**Published:** 2016-08-10

**Authors:** Cynthia Sung, Yuan Wei, Satoru Watanabe, How Sung Lee, Yok Moi Khoo, Lu Fan, Abhay P. S. Rathore, Kitti Wing-Ki Chan, Milly M. Choy, Uma S. Kamaraj, October M. Sessions, Pauline Aw, Paola F. de Sessions, Bernett Lee, John E. Connolly, Martin L. Hibberd, Dhanasekaran Vijaykrishna, Limin Wijaya, Eng Eong Ooi, Jenny Guek-Hong Low, Subhash G. Vasudevan

**Affiliations:** 1 Program in Emerging Infectious Diseases, Duke-NUS Medical School, Singapore; 2 Vigilance and Compliance Branch, Health Sciences Authority, Singapore; 3 Singapore Clinical Research Institute, Singapore; 4 Department of Pharmacology, Yong Loo Lin School of Medicine, National University of Singapore, Singapore; 5 Genome Institute of Singapore, A*STAR, Biopolis, Singapore; 6 Institute of Molecular & Cellular Biology, A*STAR, Biopolis, Singapore; 7 Department of Infectious Diseases, Singapore General Hospital, Singapore; Florida Gulf Coast University, UNITED STATES

## Abstract

CELADEN was a randomized placebo-controlled trial of 50 patients with confirmed dengue fever to evaluate the efficacy and safety of celgosivir (A study registered at ClinicalTrials.gov, number NCT01619969). Celgosivir was given as a 400 mg loading dose and 200 mg bid (twice a day) over 5 days. Replication competent virus was measured by plaque assay and compared to reverse transcription quantitative PCR (qPCR) of viral RNA. Pharmacokinetics (PK) correlations with viremia, immunological profiling, next generation sequence (NGS) analysis and hematological data were evaluated as exploratory endpoints here to identify possible signals of pharmacological activity. Viremia by plaque assay strongly correlated with qPCR during the first four days. Immunological profiling demonstrated a qualitative shift in T helper cell profile during the course of infection. NGS analysis did not reveal any prominent signature that could be associated with drug treatment; however the phylogenetic spread of patients’ isolates underlines the importance of strain variability that may potentially confound interpretation of dengue drug trials conducted during different outbreaks and in different countries. Celgosivir rapidly converted to castanospermine (Cast) with mean peak and trough concentrations of 5727 ng/mL (30.2 μM) and 430 ng/mL (2.3 μM), respectively and cleared with a half-life of 2.5 (± 0.6) hr. Mean viral log reduction between day 2 and 4 (VLR2-4) was significantly greater in secondary dengue than primary dengue (p = 0.002). VLR2-4 did not correlate with drug AUC but showed a trend of greater response with increasing Cmin. PK modeling identified dosing regimens predicted to achieve 2.4 to 4.5 times higher Cmin. than in the CELADEN trial for only 13% to 33% increase in overall dose. A small, non-statistical trend towards better outcome on platelet nadir and difference between maximum and minimum hematocrit was observed in celgosivir-treated patients with secondary dengue infection. Optimization of the dosing regimen and patient stratification may enhance the ability of a clinical trial to demonstrate celgosivir activity in treating dengue fever based on hematological endpoints. A new clinical trial with a revised dosing regimen is slated to start in 2016 (NCT02569827). Furthermore celgosivir’s potential value for treatment of other flaviruses such as Zika virus should be investigated urgently.

**Trial Registration:** ClinicalTrials.gov NCT01619969

## Author Summary

Dengue virus is currently threatening 40% of the world’s population. An approximately 60% efficacious vaccine has been registered for use in Mexico, Brazil, the Philippines, Paraguay and El Salvador, but there are no approved antiviral treatments available. We have shown that celgosivir, an endoplasmic reticulum alpha glucosidase inhibitor, has submicromolar activity against all 4 serotypes of dengue virus (DENV) and also efficacious in mouse model of infection. The strong preclinical pharmacology motivated the conduct of an investigator-initiated, Phase 1b randomized, double-blind, placebo-controlled trial of celgosivir in 50 adult dengue patients. Although the trial did not meet the primary endpoints of lowering viremia or fever, the safety profile of the drug prompted extended hematological, pharmacokinetic, immunological and viral sequence profiling. Here we report several non-significant trends of pharmacological effect of celgosivir on platelet count, hematocrit, and NS1 clearance in secondary dengue patients. In addition, pharmacokinetic modeling identified an alternate dosing regimen that is predicted to achieve a 4.5-fold increase in minimum drug concentrations during treatment (C_min_) with only a modest increase in overall dose. A new Phase 2a clinical trial with an optimized dosing regimen of celgosivir (ClinicalTrials.gov number NCT02569827) is scheduled to start in the latter part of 2016.

## Introduction

Dengue fever is a mosquito-borne viral illness that is endemic in tropical regions around the world, with an estimated 96 million cases of dengue illness annually [1]. Dengue is one of 17 neglected tropical diseases that the World Health Organization (WHO) has identified for priority attention due to its disproportionate impact on global health, with cases reported from over 100 countries [2]. Singapore maintains an aggressive mosquito control program [3], with spending by the National Environment Agency approaching US$50 million annually. These efforts have successfully driven the proportion of households harboring the Aedes mosquito, the vector for dengue, to historic lows of less than 1%. Yet, in the last decade, the incidence rates have continued to climb, with the highest rate recorded in 2013 of 404.9 cases per 100,000 with 8 deaths [4].

Currently, there are no approved drugs for dengue. Vaccine development has been underway since the 1970s [5], an extraordinarily challenging effort because immunity to one serotype does not confer protection against the others. Furthermore, a phenomenon known as antibody-dependent enhancement (ADE) posits that antibodies to one serotype from a previous dengue infection increases the risk of more serious forms of the illness, dengue hemorrhagic fever (DHF) or dengue shock syndrome (DSS) [6,7]. Indeed, the proportion of DHF among patients with secondary dengue is much higher than those with primary dengue [8,9]. Therefore, vaccine development has proceeded under the premise that a vaccine must protect against all four dengue serotypes; otherwise, potentially more serious outcomes may ensue if the subject achieves only partial immunity. Sanofi’s tetravalent dengue vaccine CYD-TDV achieved 56% and 65% efficacy in Phase 3 field studies in Southeast Asia and Latin America, respectively. Protection was serotype dependent with protective efficacy for DENV 2 of 35% and 42% in the two trials [10,11]. In December 2015, a number of countries, namely Mexico, Brazil, and the Philippines, approved CYD-TDV for use as a dengue vaccine. However, the modest vaccine efficacy, particularly in patients with no previous history of dengue infection, the higher hospitalization rate among vaccinated children younger than 9 years in the third year of follow-up [12,13], and the extended timeframe required to implement large-scale vaccination programs underscores a continuing need to discover and develop dengue drugs that can be used alongside vaccines.

Since 2008, five randomized, controlled trials of dengue drugs have been completed [14–18] All adopted a strategy of repositioning, or using drugs for which human safety data were already available from approved drugs or from trials of other clinical indications. Celgosivir, an inhibitor of alpha-glucosidase, a host enzyme required for proper processing of viral surface glycoproteins, has been given to hundreds of patients in HIV and HCV trials, but further pharma-driven development of celgosivir was discontinued because approved drugs for those indications had better or equivalent efficacy in Phase 2 trials [19,20]. When tested in cell-based assays and an animal model of dengue infection, celgosivir demonstrated submicromolar activity and prevented death in mice infected with an otherwise lethal dose of virus [21,22]. The strong preclinical pharmacology results motivated the conduct of a Phase 1b randomized, double-blind, placebo-controlled trial in 50 adult dengue patients (CELADEN, NCT01619969). Although the trial did not meet the primary endpoints of lowering viremia or fever [17], examination of data for secondary endpoints, presented here, as well as additional studies using a mouse model of infection [23] have provided insights for a new Phase 2a clinical trial with an altered regimen of celgosivir (NCT02569827). The analysis presented here may also be informative for the design of other drug trials for dengue fever.

## Methods

### Synthesis of Celgosivir

Celgosivir was synthesized according to US patent 5,017,563 [24] by selective C-acylation of castanospermine (MedChem 101 LLC). A suspension of castanospermine and bis(tributyl tin) oxide (1:2 molar ratio) in toluene (30 vol) was refluxed under argon for 3 h. The solution was cooled down to -17°C, then butyryl chloride (1.8 molar excess) was added dropwise over a 10 min period. The mixture was stirred at room temperature for 2 h. Absolute EtOH was added, and the mixture was stirred for 30 min, followed by addition of 1.5M HCl/EtOH solution (2-fold molar ratio to butyryl chloride). The mixture was stirred over night (18 h) at room temperature then for 1 h at +3°C. The precipitate was filtered, washed with hexane, and dried in vacuo. The compound was recrystallized to obtain a product with > 99% purity by HPLC. Celgosivir was synthesized, purified, capsuled in 100 mg doses, and packaged into blister packs at a GMP facility, Dalton Pharma Services (Toronto, Ontario, Canada). USP pregelatinized maize starch was prepared in identical capsules and blister packs for placebo.

### Patient Cohort

**Fig 1:**
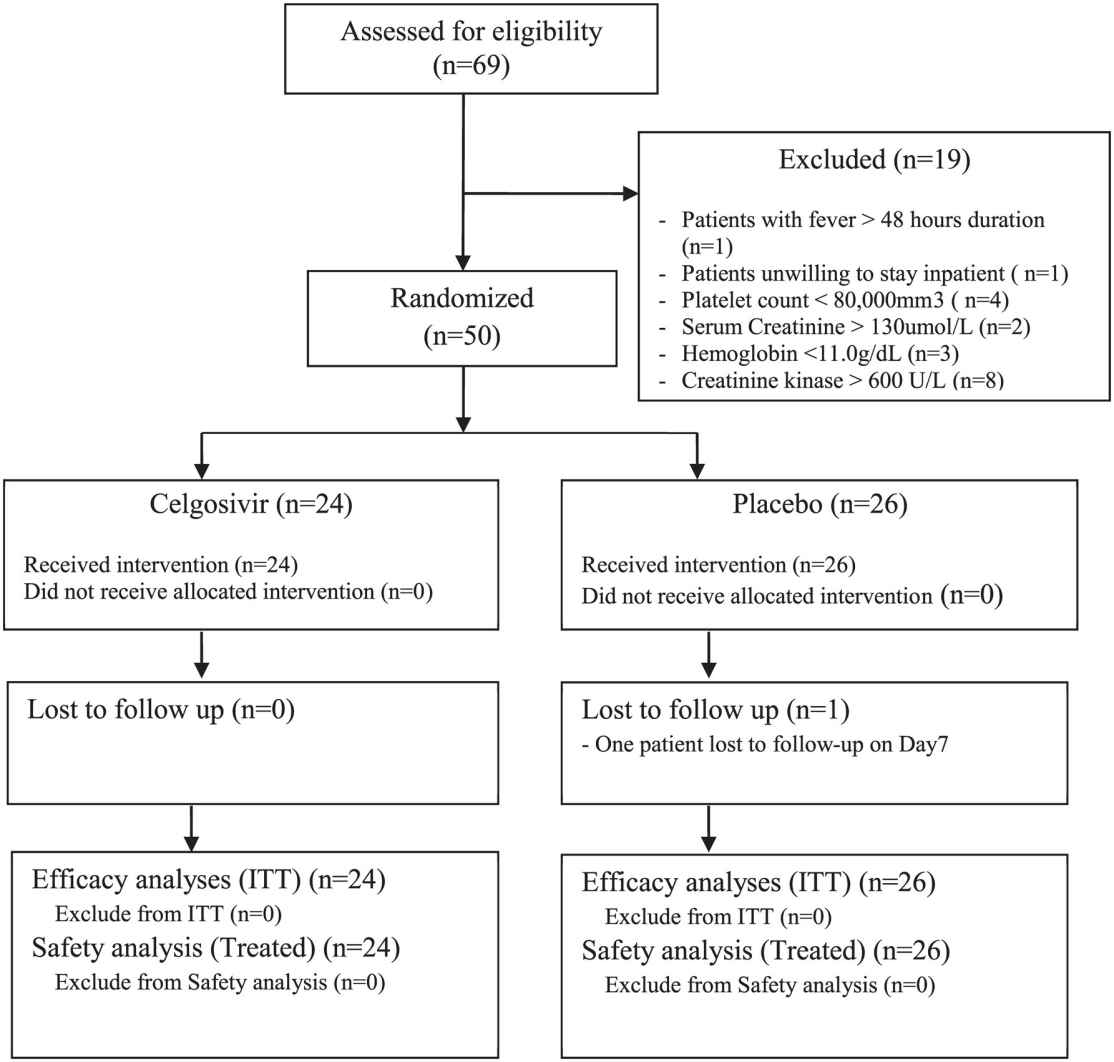
Subject disposition for CELADEN study [17].

Patient samples were obtained from a randomized, double-blind and placebo-controlled proof-of-concept trial (CELADEN) in Singapore to assess the efficacy and safety of celgosivir in patients with dengue fever [17]. The Trial Protocol is included here again as S1 Text. The Consort flowchart showing the CELADEN Trial Profile was published previously [17] and is included in this manuscript as Fig 1. The inclusion and exclusion criteria for CELADEN Trial was described previously and is included here as S1 Fig. The Dengue Duo (SD Diagnostics) point-of-care diagnostic kit was used to screen for dengue infection. It consists of two tests, one for serum NS1 and the other for dengue immunoglobulins (IgM and IgG). Fifty dengue patients identified by Dengue Duo Diagnostics and with fever >38°C for less than 48 hr were randomly assigned (24 to celgosivir, 26 to placebo), of which 14 had DENV-1, 32 had DENV-2 and 4 had DENV-3 infection. The patients were housed in the clinical trial facility at the Singhealth Investigational Medicine Unit for five days during the acute illness period and returned to the study center for follow-up examinations on study days 7, 10 and 15. Immunoglobulin M antibody capture or dengue IgG indirect ELISA (Panbio Diagnostics, Providence RI) were performed on baseline samples to identify primary and secondary infection status as described previously [17].

### Ethics Statement

The study was approved by the Singapore Health Services’s Centralized Institutional Review Board (CIRB Ref:2012/025/E)—and monitored independently by the Singapore Clinical Research Institute (SCRI), a publicly funded clinical research organization (CRO) [17]. All subjects between the ages of 21–65 years provided written consent to participate in the inpatient trial as described in detail in our previous publication [16]. It should be noted that all deviations to the study progress during the trial was provided to the Health Sciences Authority of Singapore and also to an independent data safety monitoring board. The CELADEN trial was registered with www.ClinicalTrials.gov, number NCT01619969.

### Viremia, Clinical Chemistry and Hematology

The method for determination of viral RNA by quantitative polymerase chain reaction (qPCR) is described elsewhere [17, 22]. Viral load reduction (VLR) was defined as the difference between viremia (determined by qPCR) at enrollment and at each study day. The primary endpoint was the mean VLR between study days 2 and 4 inclusive (VLR2-4), which is mathematically equivalent to the area under the curve (AUC) of the VLR curve between those days divided by the number of days. A plaque assay was also performed to measure viremia, as described in [22,25]. The plaque assay measures the number of virus particles capable of productive infection, unlike the qPCR assay that measures RNA of all viral particles, regardless of replication competency. Hematology and clinical chemistry assays were standard assays performed in the clinical diagnostic laboratories of Singapore General Hospital.

### ELISA for Dengue IgG

A sandwich-based ELISA assay using DENV-2 protein for capture and goat anti-human antibody conjugated with horseradish peroxidase as the detecting antibody. Patient sera and positive and negative human control sera were diluted 1:100 in serum diluent prepared using PBS with 0.5% nonfat dry milk. The plates were developed with tetramethylbenzidine as the substrate [26].

### Pharmacokinetics

PK samples were collected prior to the first dose and at 23, 25, 47, 49.5, 71, 74 and 95 hr after the first dose, representing 4 trough and 3 peak levels. Urine was collected in 12-hr periods, the volume recorded, and a sample reserved for drug assays. Celgosivir and castanospermine concentrations were determined on a LC/MS/MS system (Hewlett Packard 1100 with Applied Biosystems API 3200 MSMS). N-dodecyl-deoxynojirimycin was the internal standard for celgosivir, and 6,7 dihydroxyswainsonine was the internal standard for castanospermine. The LC column was a Waters Atlantis HILIC Silica column; the mobile phase consisted of 23% 20 mM Ammonium acetate pH 5.0 in pump A and 77% acetonitrile in pump B for 7 min then changed to 60% in pump A for 3.9 min. The LC eluent was connected directly to a Sciex API 3200 triple-quadrupole MS equipped with electrospray ionizing ion source without splitting. The quadrupoles were operated with unit resolution in the positive ion multiple reaction monitoring mode. The assay had a lower limit of quantitation (LLoQ) of 10 ng/mL and a linear response over the range of 10 to 2000 ng/mL.

Concentration-time profiles were analyzed with Phoenix WinNonlin v6.3 using a one-compartment model with first-order absorption and elimination (Model 3) and weighting by the inverse of predicted concentration. Two samples were excluded from PK analysis: one was a trough sample that had a concentration nearly 5 times higher than the other trough samples from the same patient. The other was a peak sample that had a concentration more than 10 times lower than the other peak samples from the same patient. Both concentrations were more than 3 standard deviations from the mean of the other patients’ samples at the same time point. Body weight, age, sex, and renal clearance were evaluated as covariates. Renal clearance was estimated from serum creatinine levels, patient demographics, and the Crockcroft-Gault formula. We performed simulations of several dosing regimens being considered for a follow-on trial of celgosivir in adult dengue subjects. These were performed with Model 3 using fitted parameters for the population of patients in the CELADEN trial.

The primary virological endpoint of the trial was the mean virological log reduction between study days 2 and 4 (VLR2-4). To explore the relationship between VLR2-4 and PK, patients’ exposure (Cmin, Cmax or AUC) was subdivided into 3 quantiles: zero exposure (placebo), low drug exposure (lower quantile) and high drug exposure (upper quantile), and the distribution of VLR2-4 in each group was graphed.

### Cytokine Multiplex Analysis

Patient plasma samples, drawn at 24, 48, 72 and 120 hr after the first dose, were analyzed for 41 cytokines and chemokines using the Human cytokine panel 1 (Merck Millipore cat no. MPXHCYTO-60K-14) as per manufacturer’s protocol. Briefly, samples were diluted 1:2.5 with RPMI + 10% fetal calf serum and loaded onto a Millipore Multiscreen BV 96-well filter plate. Serial dilutions of cytokine standards were prepared in parallel and added to the plate. Milliplex Cytokine beads were vortexed for 30 sec. and 25 μl was added to each well with culture supernatants. Samples were then incubated on a plate shaker at 600 rpm in the dark at room temperature for 2 hr. The plate was applied to a Millipore Multiscreen Vacuum Manifold, washed twice with 50μl of assay buffer (PBS, pH7.4, 1% BSA, 0.05% Tween20, 0.05% sodium azide), and each well resuspended with 75μl assay buffer. Twenty-five μl of biotinylated Anti-Human Multi-Cytokine Reporter was added to each well. The plate was incubated on a plate shaker at 600rpm in the dark at room temperature for 1.5 hr. Streptavidin-Phycoerythrin was diluted 1:12.5 in assay buffer, and then 25μl was added directly to each well. The plate was again incubated on a plate shaker at 600rpm in the dark at room temperature for 30 minutes. Twenty-five μl of stop solution (0.2% (v/v) formaldehyde in PBS, pH 7.4) was added to each well and incubated at room temperature for 5 minutes. The plate was then applied to the vacuum manifold and each well resuspended in 125μl assay buffer and shaken for 1 minute. Assay plates were read with Flexmap 3D systems (Luminex Corp, Austin, TX, USA). Cytokine concentrations were calculated using Bio-Plex Manager 6.0 software with a 5 parameter curve fitting algorithm applied for standard curve calculations.

### Statistical Evaluation of Viral Correlations and Secondary Endpoints

Correlations between log10 plaque forming units (pfu) and log10 viral RNA copy numbers measured using qPCR at each day were evaluated by the Pearson correlation coefficient. Fisher’s exact test was used to compare the number of negative plaque assays (zero pfu) between celgosivir and placebo groups by day and between primary and secondary dengue. Student’s t-test for two independent samples was used to compare VLR2-4 between primary and secondary dengue. The t-test was also used to compare celgosivir and placebo-treated secondary dengue patients’ platelet nadirs and differences between the maximum minus the minimum hematocrit. Covariate dependencies on PK parameters were evaluated by linear regression and deemed statistically significant if the 95% confidence interval of the slope excluded zero. The Kruskal-Wallis test was performed to evaluate the relationship between exposure (quantiles of Cmin, Cmax, and AUC) and VLR2-4. Two-way repeated ANOVAs were used to analyze the effects of time and treatment on the Luminex analyte concentrations. Graphing and statistical evaluations were performed with R version 2.15.2, Graphpad Prism v 5.0d or SAS statistical software.

### Whole-Genome Amplification and Next-Generation Sequencing of DENV Isolates

Next-generation whole-genome sequencing of DENV samples isolated from blood at study days 1, 2, 3 and 4 was performed as described previously [27, 28]. Viral RNA was extracted from human sera using the QIAamp Viral RNA Mini Kit (Qiagen), and cDNA synthesis for each serotype was carried out with the Maxima H Minus First Strand cDNA Synthesis Kit (ThermoFisher Scientific) using serotype-specific primers designed to bind to the 3’ end of the viral genome. The entire DENV genome was PCR-amplified in 6 overlapping fragments, each approximately 2 kb in length with the PfuUltra II Fusion HS DNA Polymerase (Agilent Technologies). Table 1 lists the number of patient samples that were extracted and Table 2 lists the primer sequences used for the dengue serotypes. PCR products were gel-extracted and purified using the Qiagen Gel Extraction Kit (Qiagen).

**Table 1:** Patient sample information.

*Serotype*	*Treatment*	*Number of sequences obtained from patients*
*DENV1*	*Placebo*	*4*
	*Celgosivir*	*6*
*DENV2*	*Placebo*	*17*
	*Celgosivir*	*8*

**Table 2:** Sequences of primers used to amplify the DENV1 and DENV2 genome in 6 overlapping fragments.

*DENV1 Fragment*	*Forward (5'-3')*	*Reverse (5'-3')*	*Region*
*1*	*AGTTGTTAGTCTACGTGGAC*	*ACACCGCTGAACAAAACTCC*	*1–2084*
*2*	*TCACAAGAAGGAGCAATGCACA*	*AAGAAGAACTTCTCTGGATGTTA*	*1697–3784*
*3*	*ACCAATGTTTGCTGTAGGGC*	*TATTCCCCGTCTATTGCTGC*	*3727–6090*
*4*	*CAGAGCAACGCAGTTATCCA*	*CAATTTAGCGGTTCCTCTCG*	*5501–7759*
*5*	*TCACAGATCCTCTTGATGCG*	*CATGGCACCACTATTTCCCT*	*7364–9753*
*6*	*ATGGCTCACAGGAAACCAAC*	*TGCCTGGAATGATGCTGTAG*	*8303–10690*
*DENV2 Fragment*	*Forward (5'-3')*	*Reverse (5'-3')*	*Region*
*1*	*AGTWGTTAGTCTACGTGGAC*	*TGGGCTGTCTTTTTCTGTGA*	*1–2028*
*2*	*GCAGAAACACAACATGGAACA*	*AACGCGTCAGTCAGTTCAAG*	*1873–3857*
*3*	*GAAAGCTGACCTCCAAGGAA*	*CTGAAATGTCTGTCGTGACCA*	*3578–5755*
*4*	*AGGCAGCTGGGATTTTCATGA*	*TTTCCCTTCTGGTGTGACCA*	*5444–7797*
*5*	*ACTCAAGTATTGATGATGAGGA*	*TGTGTCCAATCGTTCCATCCT*	*7360–9683*
*6*	*GCAGGATGGGACACAAGAAT*	*AGAACCTGTTGATTCAACAG*	*9175–10703*

For each sample, equal amounts of all PCR-amplified fragments were combined and sheared on the Covaris S2 sonicator (Covaris) to achieve a peak size range of 100–300 bp (shearing conditions: duty cycle—20%; intensity—5; cycles per burst—200; time—110 seconds). Samples were purified with the Qiagen PCR Purification Kit (Qiagen) and their quality assessed on the Agilent 2100 Bioanalyzer with a DNA 1000 Chip (Agilent Technologies). Library preparation was performed with the KAPA Library Preparation Kit (KAPA Biosciences). After end-repair, A-tailing, and adapter ligation, ligated products in the 200–400 bp range were gel-extracted with the Qiagen Gel Extraction Kit (Qiagen). Samples were subjected to 14 PCR cycles to incorporate multiplexing indices and quantified using the Agilent Bioanalyzer. Samples were then diluted to 10 nM and pooled. Paired-end multiplexed sequencing (2 x 76 bp reads) [29] of libraries was performed on the Illumina HiSeq (Illumina) at the Genome Institute of Singapore.

### Mapping and Single Nucleotide Variant (SNV) Calling

FastQC [FastQC: A quality control tool for high throughput sequence data (http://www.bioinformatics.babraham.ac.uk/projects/fastqc/)] was used to check the quality of the reads from Illumina-generated FASTQ files. Trim Galore! was used to trim and filter the reads with minimum quality cutoff of 20 and minimum read length of 35 bp. The consensus genome for the sample at time point one was generated using the bam2cons_iter.sh script from the Viral Pipeline Runner (ViPR, available at https://github.com/CSB5/vipr), which uses the Burrows-Wheeler Aligner to perform iterative mapping of paired-end reads to the reference [29]. DENV genome of samples isolated from later time points was then mapped against the consensus, generated based on the maximum frequency of the nucleotide at a given position, using BWA-MEM v0.7.5 aligner. Picard Tools v1.95 [30] were used to remove PCR duplicates and base calibration, and indel realignment was done by GATK v3.3. SNVs for each sample were detected using LoFreq2 software [31], which incorporates base-call quality scores as error probabilities into its model to distinguish SNVs from the average sequencing error rate, and assigns a p-value to each position (Bonferroni-corrected p-value > 0.05). As LoFreq has previously been applied to DENV datasets, and its SNV predictions on these datasets have been experimentally validated down to 0.5% allele frequency [27, 32], we filtered the SNPs with a threshold of coverage of >1000 and allele frequency of >0.5%. SNVs that were located within primer sequences were discarded. An in-house R script was used to group the samples and count the number of SNVs occurring at each genomic position. The dN/dS analysis, mutation density (SNVs per 100 bp) and all statistical tests were also performed in R. For identification of mutational cold spot, SNVs from groups of samples were pooled and then scanned for windows (minimum size of 40) with a depletion of SNVs (binomial test; Bonferroni corrected p-value < 0.05).

### Phylogenetic Analysis of Virus Genomes

Full genomes of DENV strains isolated from treatment and placebo patients were analyzed for each DENV type independently, along with DENV genomes originating from Asia that were retrieved from NCBI GenBank. Multiple sequence alignments were performed using MAFFT [33]. Maximum likelihood phylogenetic trees were constructed using RAxML applying the General time reversible (GTR) model with gamma distributed rates across sites (GTR+γ) [34]. Trees were visualized and annotated using FigTree v1.5 (http://tree.bio.ed.ac.uk/software/figtree/).

## Results

### Quantification of Viremia

**Fig 2:**
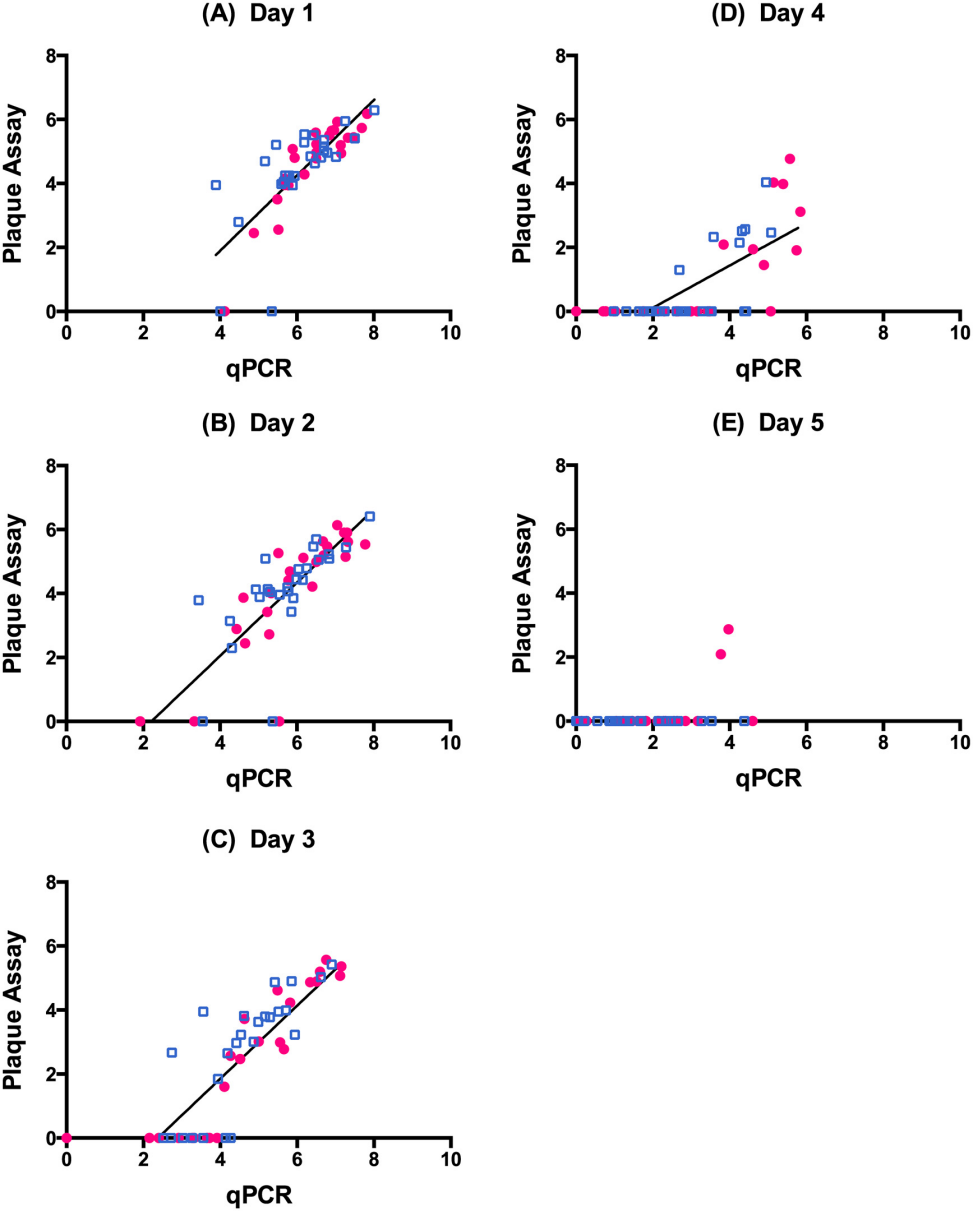
Correlation between viremia by qPCR and plaque assay. Red filled circles–patients who received celgosivir; blue open squares–patients who received placebo; lines–linear regression to all data. Pearson correlation coefficient for all data (A)Day 1 0.79 (95% CI: 0.64 to 0.87); (B) Day 2 0.79 (95% CI: 0.65 to 0.87); (C) Day 3 0.83 (95% CI: 0.72 to 0.90); (D) Day 4 (95% CI: 0.55 to 0.83); (E) Day 5 0.36 (95% CI: 0.08 to 0.58).

**Fig 3:**
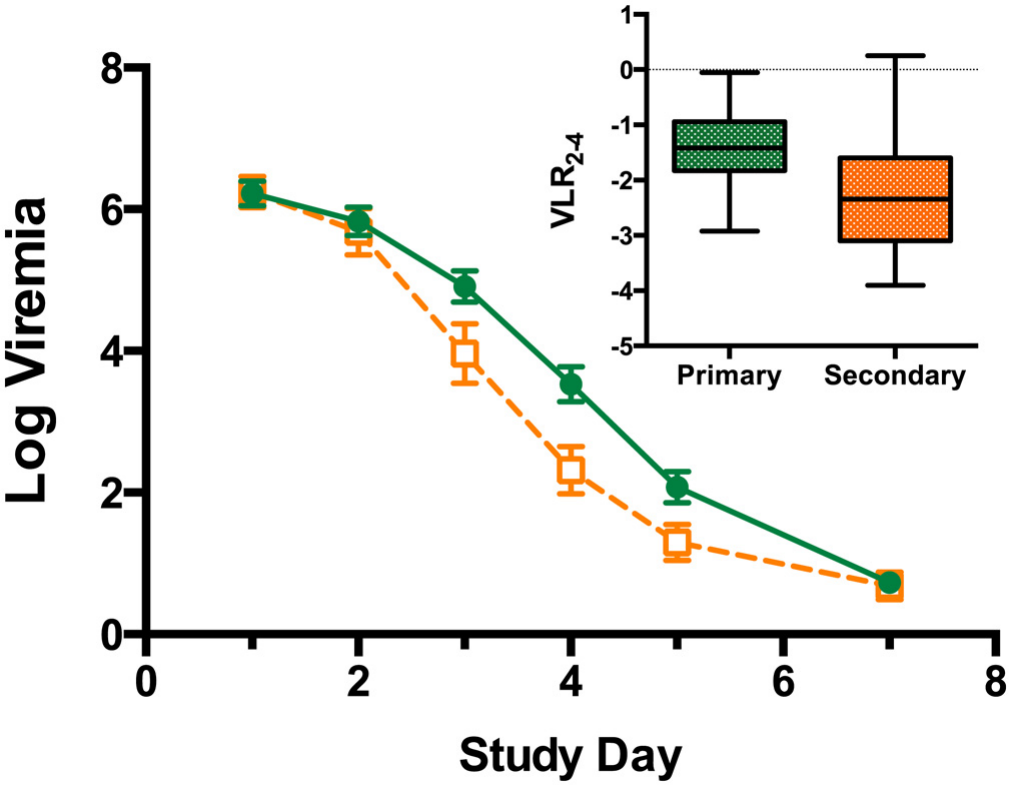
Viremia kinetics in primary and secondary dengue patients. Log viremia Mean (± SEM) by day and prior dengue infection status. Green solid circles connected by solid line–primary dengue; open orange squares connected by dashed line–secondary dengue. Inset: VLR 2–4 by prior dengue infection status: box 25th to 75th percentile, whiskers minimum and maximum values. Virus is cleared significantly faster in secondary dengue compared to primary dengue (p = 0.002).

All the 50 patients recruited to the study who tested positive on the Dengue Duo NS1 screening kit and recruited to the study had virologically confirmed dengue [17] (Fig 1). On days 1, 2 and 3, there was strong and significantly positive correlation between viremia measured by qPCR and the plaque assay (Fig 2A–2C) although by day 3, only 66% of the samples had positive viremia by the plaque assay. By day 4, the correlation was weaker, but still significant (Fig 2D), with only 34% of samples having positive viremia by the plaque assay compared to 98% by qPCR. By day 5, only two samples were positive for dengue virus by plaque assay (Fig 2E). There was no treatment effect when comparing the frequency of negative plaque assay results for celgosivir and placebo groups. The kinetics of viral clearance (qPCR) was faster in secondary dengue than in primary dengue (Fig 3). VLR2-4 for patients with secondary dengue (-2.25 ± 1.04) was significantly lower (p-value 0.002) than for those with primary dengue (-1.46 ± 0.70); the difference (95% CI) was 0.79 (0.30, 1.29). On days 3 and 4, there were a significantly higher number of negative plaque assays in secondary dengue patients compared to primary dengue patients (Day 3: p-value<0.001, OR = 10.8, 95% CI 2.75 to 42.4; Day 4: p-value = 0.029, OR = 5.79, 95% CI 1.13 to 29.6).

### Pharmacokinetics

**Fig 4:**
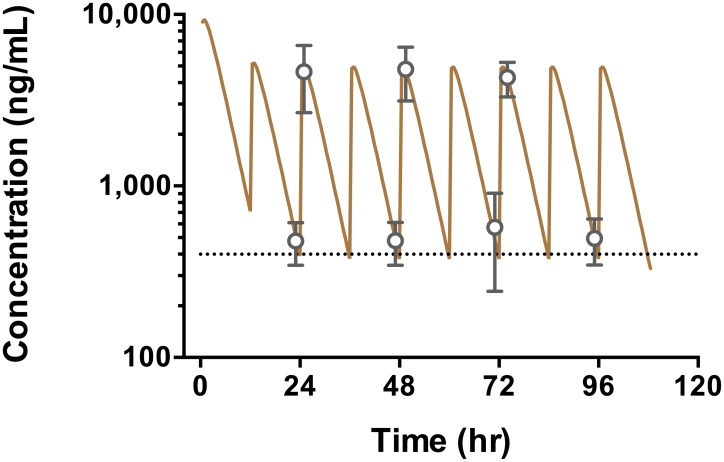
PK profile of castanospermine (semi-log plot). Solid brown line is the predicted concentration of castanospermine based on the mean PK parameters and the dosing regimen studied in the trial. Gray open circles are the observed peak and trough concentrations of castanospermine. Symbols and error bars are the mean and SEM, respectively. Black dotted line is the target trough concentration (400 ng/mL) predicted based on animal efficacy studies.

**Fig 5:**
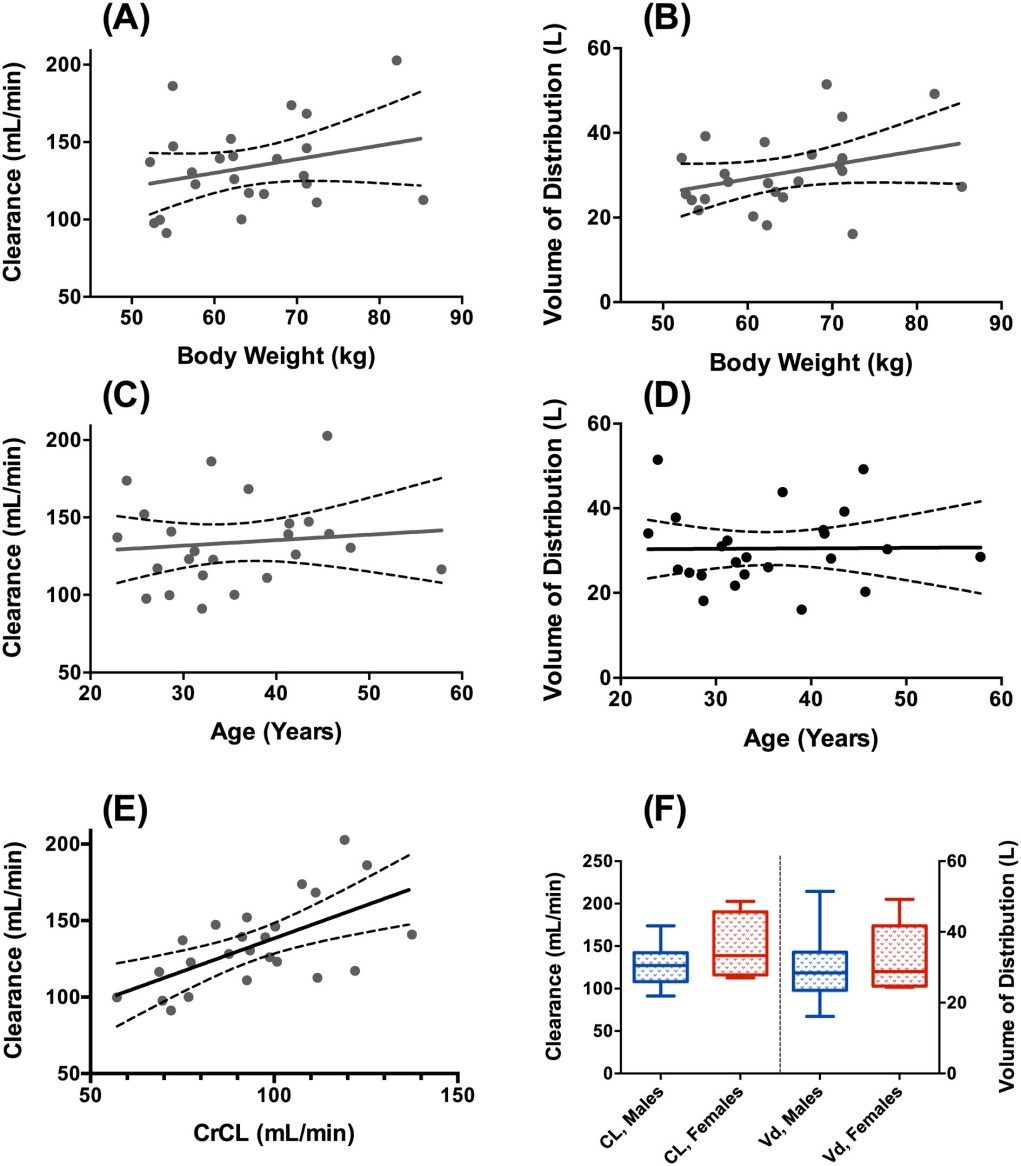
Dependence of pharmacokinetic parameters on covariates. Body Weight (A and B); Age (C and D); Creatinine Clearance (E); and Sex (F). Clearance or volume of distribution were not significantly affected by patients’ body weight, age or sex. Drug clearance was significantly correlated with creatinine clearance, indicating a significant role of the kidneys for elimination of celgosvir. Solid line-linear regression, dashed line- 95% CI. The slope of the linear regression line of creatinine clearance versus drug clearance was 0.86 (95% CI: 0.376, 1.351).

Celgosivir was rapidly converted to castanospermine in vivo, presumably by endogenous esterases, as expected from previous animal and human studies [19]; 83% of the samples had no quantifiable levels of parent drug above the lower limit of quantification. Observed mean castanospermine Cmin and Cmax were 430 ng/mL (2.23 μM) and 5730 ng/mL (30.2 μM), respectively (Table 2). Mean (± sd) oral clearance (CL/F) was 132 (± 28) mL/min. The mean volume of distribution (V/F) was 28.2 (± 9.1) L, and half-life was 2.5 (± 0.6) hr. Using PK parameters from compartmental modeling, the predicted mean plasma concentration profile during the entire dosing period is shown in Fig 4. The data showed that the actual drug concentrations remained above 400 ng/mL during the dosing period when mean viremia levels started from greater than 6 logs and declined by more than 4 logs (Fig 3). The target concentration of 400 ng/mL was the trough concentration in mice treated with celgosivir using a dosing regimen (50 mg/kg bid for 5 days) that protected all animals from otherwise lethal dengue infection [22]. Body weight, age and sex were not significant covariates on clearance or volume of distribution (Fig 5A–5D and 5F). On the other hand, castanospermine clearance and renal (creatinine) clearance were significantly correlated (Fig 5E). Drug in the urine was >99% castanospermine, and urinary recovery was 80% ± 17%.

**Fig 6:**
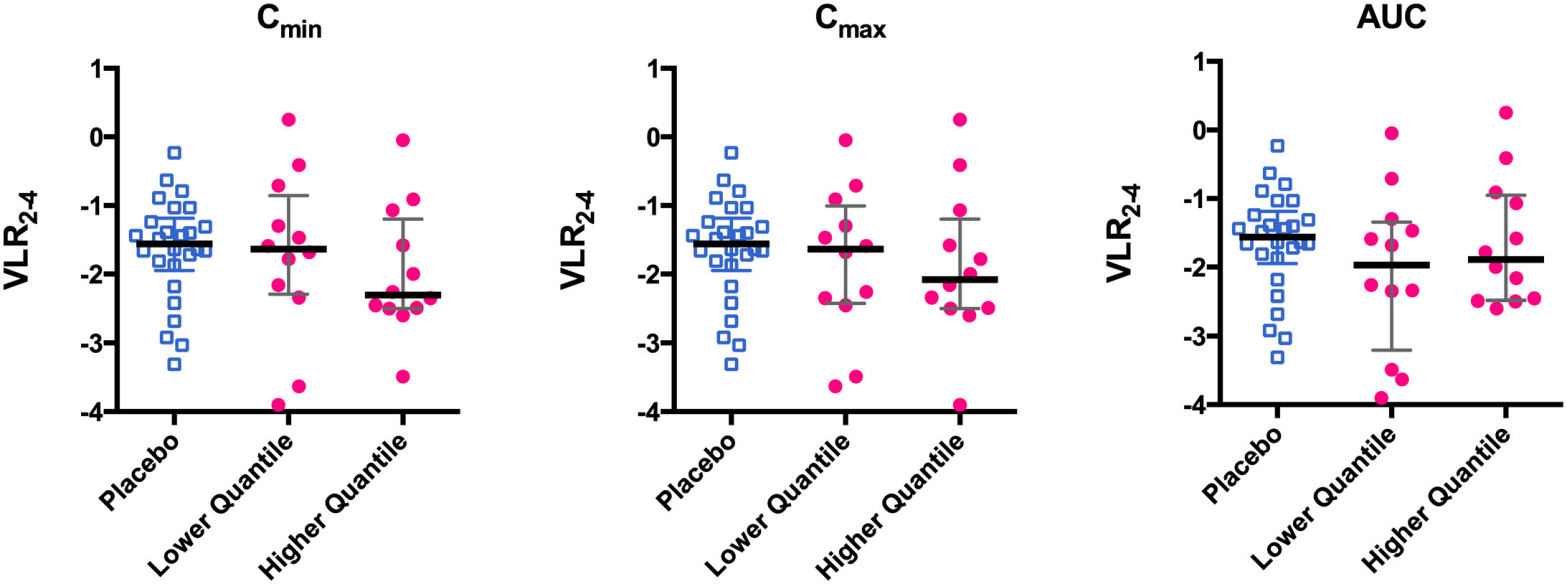
Scatterplots of drug exposure (Cmin, Cmax, AUC) and VLR2-4. Blue open squares–individual patients who received placebo; red filled circles–individual patients who received celgosivir, separated into two quantiles of exposure. solid heavy line–median value, error bars– 25th to 75th percentile.

Although only one dose was evaluated, a 3-fold range of exposure was obtained in observed Cmin and a two-fold range in Cmax and predicted AUC (Table 3). There was a subtle trend for lowered viremia (VLR2-4) with increasing Cmin (Fig 6) that was not evident with AUC. This is in line with the concept of maintaining a serum concentration above a minimum inhibitory concentration for optimizing antimicrobial/antiviral drug therapy. Although there was a trend between VLR2-4 and quantiles of exposure of Cmin, and Cmax, neither one was significantly correlated (Fig 6).

**Table 3:** Castanospermine PK parameters.

	C_min,obs_	C_max,obs_	CL/F	V/F	K_01_	AUC_ss_	T_max_	T_1/2_
	ng/mL	ng/mL	mL/min	L	1/hr	ng/mL*hr	hr	hr
Mean	**430**	**5727**	**131.8**	**28.2**	**4.48**	**26306**	**1.23**	**2.48**
SD	116	1175	27.9	9.1	4.76	5154	0.77	0.57
Min	225	3970	92.6	15.3	0.4	16546	0.28	1.52
Median	412	5625	128.1	26.2	1.99	26017	1.21	2.71
Max	694	8500	201.5	49.9	14.94	35989	2.41	3.33
CV%	26.9	20.5	21.2	32.1	106.4	19.6	62.3	22.9

**Fig 7:**
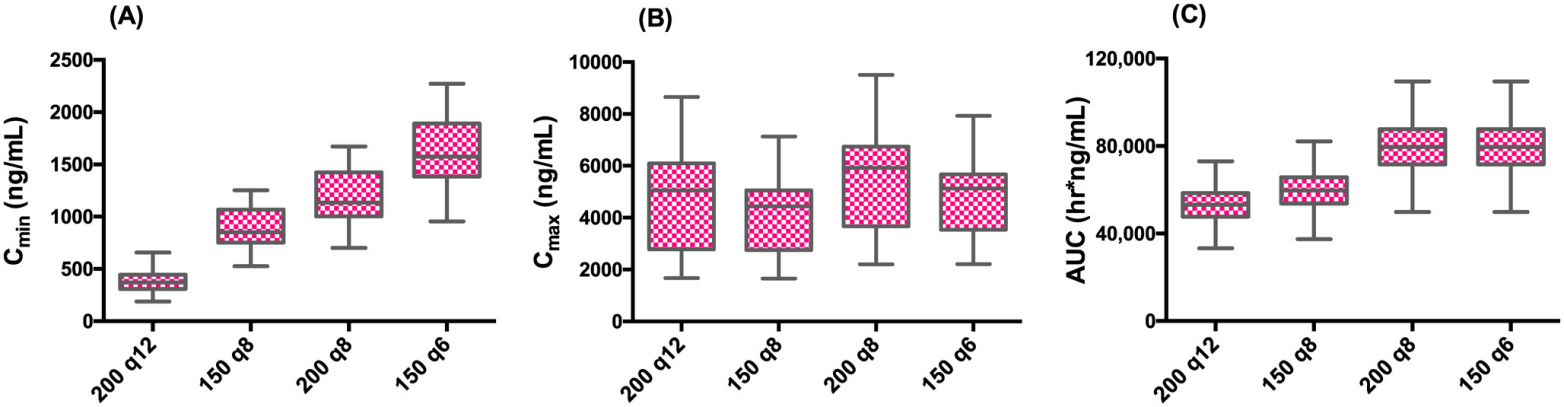
Predicted exposure for different dosing regimens. The Box-25th to 75^th^ percentile, whiskers-minimum and maximum values for the various dosing regimens is shown. (A) C_min_ range for the various dosing regimens shows that 150 mg every 6 hr is predicted to yield a 4.5-fold increase in median Cmin used in CELADEN trial (B) C_max_, range do not vary significantly for the various dosing regimens and (C) AUC only shows a modest 1.33-fold increase over the dosing regimen used in the CELADEN trial.

The PK parameters obtained from model fitting were used to predict Cmin, Cmax and AUC for other dosing regimens (Fig 7). Simulations predicted that 150 mg given 8 hourly (total daily dose of 450 mg) would increase steady-state Cmin by 2.4-fold, decrease steady-state Cmax by 20% and increase daily AUC by only 13% compared to the regimen used in CELADEN. Doses of 200 mg every 8 hr or 150 mg every 6 hr (total daily dose of 600 mg) would achieve increases in steady-state Cmin by 3.2- and 4.5-fold, respectively with only a modest 33% increase in daily AUC.

### Hematological, Immunomonitoring and NGS Analysis

**Fig 8:**
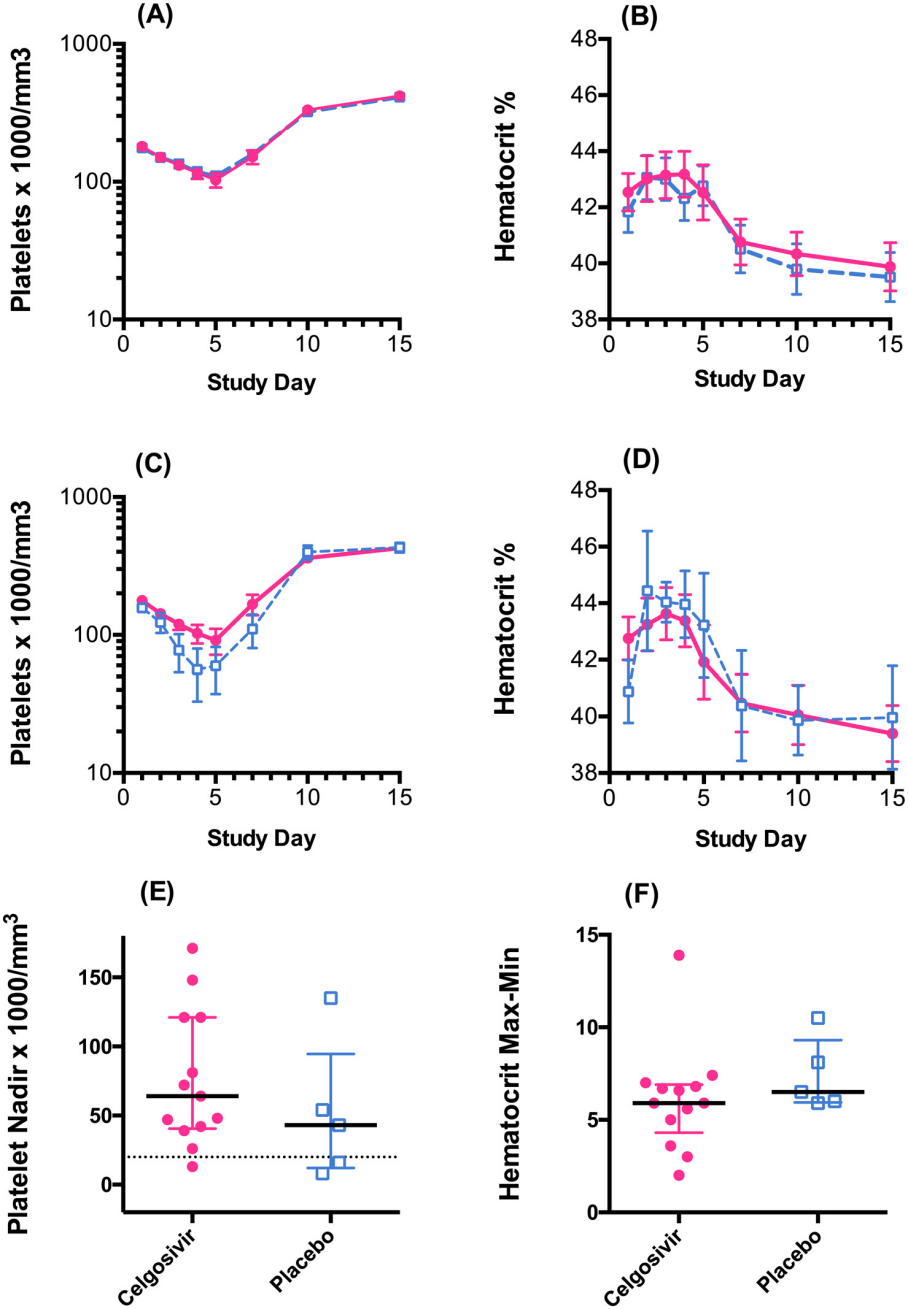
Changes in platelets and hematocrit for celgosivir (red filled circles) or placebo (blue open squares) treated patients. Mean ± SEM changes in platelets count (A) and hematocrit (B) at different study days in all patients. (C) and (D) are Mean ± SEM changes in platelets count (C) and hematocrit (D) at different study days for secondary dengue patients only. Platelet nadir values (E) and difference between maximum and minimum hematocrit values (F) for secondary dengue patients treated with celgosivir or placebo, solid line–median, bars—interquartile range.

The profiles for platelet count and hematocrit in the celgosivir and placebo groups are illustrated in Fig 8A and 8B. These show that the curves for the treatment and control groups are almost exactly superimposed. However, because the celgosivir group had a much higher proportion of secondary dengue (13/24 = 54%) than the placebo group (5/26 = 19%), and secondary dengue patients typically have a greater decrease in platelets and higher increase in hematocrit (7, 8, 21 and 22), the comparable profiles are suggestive that celgosivir had some benefit in secondary dengue. When only secondary dengue cases were compared, a trend toward better outcomes in the celgosivir treated group is discernible (Fig 8C and 8D). Platelet nadir and the difference between the maximum and minimum hematocrit for secondary dengue patients are shown in Fig 8E and 8F. The differences were not significant but are in the direction of benefit for celgosivir. Due to the small numbers of patients in this subgroup, caution is warranted not to over-interpret these trends.

**Fig 9:**
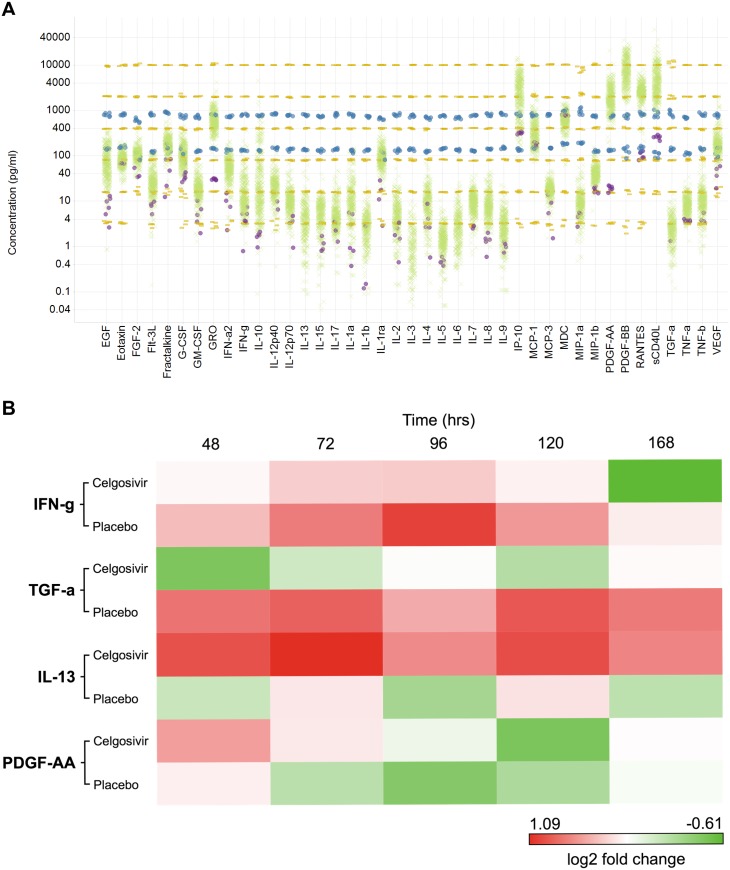
Celgosivir treatment changes the systemic immune response during acute dengue infection. A) Scatter plot of cytokine concentration of patient plasma samples drawn at specific time points post admission were analyzed for 41 cytokines and chemokines using the Human cytokine panel 1. The standard for each analyte between 4–10000 pg/ml is presented as yellow dashes, quality control for each analyte is in blue filled circles, a healthy volunteer (JEC) sample is shown in black filled circles the patient samples (400 samples/analyte; 8 per patient x 50 patients) are shown as green crosses. B) Heat map of log2 fold changes of averaged analyte concentrations at specific time points against the pre-treatment concentrations for selected analytes showing qualitative shifts in the analyte concentrations. Treatment and control groups fold changes are shown in separate rows for each analyte to aid comparison. Fold changes are colored from over expression in red to under expression in green. A scale showing the value of the maximum and minimum log2 fold change is shown.

Numerous studies have demonstrated that the magnitude and quality of systemic immune response during febrile dengue illness is linked to pathological disease progression. It has been hypothesized that the expression of proinflammatory cytokines from innate and adaptive immune effector cells plays a critical role in the cell activation, apoptosis, and vascular permeability characteristics of dengue hemorrhagic fever [35, 36]. A comprehensive analysis of circulating cytokines and chemokines was undertaken to assess the systemic impact of celgosivir treatment on the immune status of acutely infected patients. Fig 9A shows the concentration of circulating analytes at all time points for all patients in the trial. Longitudinal analysis of plasma cytokine concentrations (Fig 9B) demonstrated that drug treatment led to a qualitative shift in circulating cytokine and growth factor concentrations during the course of infection. Significant increases in IL-13 and PDGF-AA concentrations were observed in celgosivir-treated patents relative to placebo-treated patients indicating an increase in Th2 polarizing cytokines with time in this group. In support of this interpretation, drug treatment led to a corresponding decrease in circulating levels of IFNγ and TGFα. This qualitative shift from a Th1 to Th2 profile in patients receiving treatment may be reflective of a larger shift in T-cell polarization during the course of treatment as observed in other antiviral treatments [37].

Due to the limited number of samples for DENV3, NGS analysis was performed only on DENV1 and DENV2 isolates. Overall, our deep sequencing data shows positional variance throughout the DENV genome for placebo and celgosivir-treated patients (S2 and S3 Figs). Single nucleotide variants (SNVs) in each DENV population were called with the LoFreq variant calling algorithm. As in other studies [28,31], the majority (80% average across all samples) of SNVs identified in our data set were transitions (S4 Fig). For both DENV1 and DENV2, the bulk of the SNVs detected were present evenly in both treated and untreated populations, indicating some level of non-specific selective pressure. However, significant difference in selection pressure or genetic drift was observed between the different treatments for any of the viral genes (S1 Table). Interestingly, the average SNV density for each dengue gene was lower for celgosivir-treated patients compared to placebo-treated control (S2 Table).

No mutational hotspots were detected in any of the conditions for both DENV serotypes. Consistent with a previous DENV1 study [32], cold-spots were detected in NS3 of DENV1 from placebo-treated samples (P-tp3). More cold-spots were also detected for celgosivir-treated samples (C-tp1 and C-tp2) than placebo samples (P-tp1 and P-tp2) for DENV1 (S5A Fig). For DENV2, coldspots were detected mostly in NS3 and NS5 for placebo-treated samples. For celgosivir-treated samples, coldspots show a different profile from that of DENV1 and only exists in the NS5 region (C-tp1 and C-tp3) (S5B Fig).

### Phylogenetic Relationships of DENV Strains

**Fig 10:**
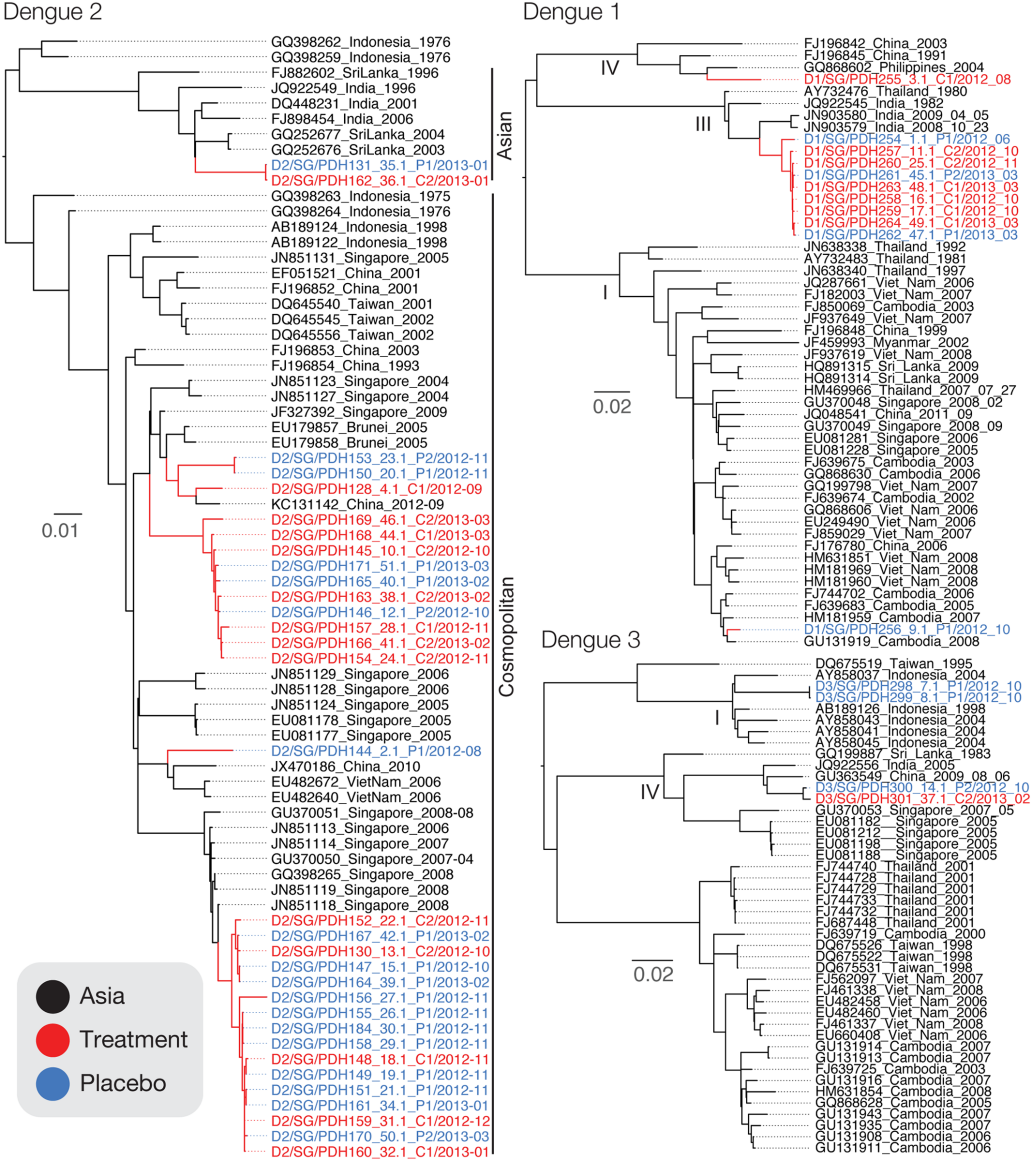
Evolutionary relationship of the whole genome of dengue samples isolated from celgosivir treatment and placebo in relation to representative samples collected from Asia. Maximum likelihood trees were generated using the nucleotide alignment from start to stop codon of the coding region. Tip labels are coloured based on sample type, and genotypes are labelled adjacent to tip labels for DENV 2, and along branches for DENV 1 and 3. Scale bar represents nucleotide substitutions per site.

Phylogenetic analysis of the whole genome sequences of treatment and placebo samples revealed the co-circulation of multiple DENV lineages in our study (Fig 10). In particular, for each DENV serotype, the treatment and placebo samples were derived from multiple genotypes. Five independent lineages of DENV 2 were detected in this study, which belonged to two major DENV 2 genotypes (Cosmopolitan and Asian). While the majority of DENV 2 samples were of the Cosmopolitan genotype we observed that they were derived from lineages that have diverged many years ago, however both these lineages have been previously detected in Asia during 2004–2012. Two lineages of DENV 1 and DENV 3 were also detected in our samples, respectively, that belonged to different genotypes. Samples obtained from treatment and placebo patients were inter-dispersed among all lineages–represented by blue and red labeled strains in each of the lineages, except in DENV 1 genotype IV and DENV 3 Genotype I where only one sample type was detected (Fig 10). We were unable to assess the statistical significance of sample type for each of the lineages due to small samples numbers among the lineages. However, these results suggest that the high genetic diversity of dengue may be a confounding factor in the endpoint analysis of this drug trial. Currently, it is not known if the genetic changes prevalent between the different lineages would have an effect on the action of celgosivir, or related anti-dengue drugs in early phase development such as UV-4, which also target ER alpha glucosidases [38].

## Discussion

Although CELADEN did not meet its primary endpoint of lowering viremia or fever, assessment of the PK and pharmacodynamics provides valuable insights and lessons for the design of future dengue drug trials, not only of celgosivir but of other dengue antivirals as well. An important objective of early phase clinical trials is to identify dose regimen(s) that are safe and tolerable and that show some evidence of pharmacological activity. Previous clinical experience in hundreds of subjects (healthy volunteers, HIV and HCV patients) established that celgosivir’s maximally tolerated dose (MTD) is 400 mg qd (once a day) for 12 weeks [20]. A small trial in HCV patients (N = 43) reported asymptomatic, reversible increases in creatine kinase (19% for 200 mg qd, 42% for 200 mg bid and 80% for 400 mg qd), suggesting that a divided dose would be better tolerated than a single daily dose of the same amount of total drug [19]. Among CELADEN patients who received celgosivir, the mean observed trough castanospermine concentration was 430 ng/mL (2.3 μM). This was comparable to the corresponding Cmin in the mouse model (400 ng/mL) where 50 mg/kg bid for 5 days protected all animals from dengue-related death when treated immediately after infection, and was far more effective than 100 mg/kg qd [22]. When treatment in mice was delayed by 24 and 48 hr post-infection, survival rates were lower at 75% and 50%, respectively [21]. In dengue patients, symptoms do not arise until several days after infection. Therefore, it may be necessary to achieve higher trough drug concentrations to overcome the delay in treatment after becoming infected. By decreasing the dosing interval from 12 to 8 or 6 hr, PK simulations demonstrate that 2.4- to 4.5-fold increase in Cmin are achievable with only a 13% to 33% increase in total dose. Indeed, AG129 mice treated at peak viremia with a four-times-daily regimen had a significantly reduced viremia compared to untreated animals in a mouse viremia model using clinical isolates of DENV2 [23].

Drug clearance was significantly correlated with creatinine clearance, and 80% of the drug was recovered in the urine, indicating that renal clearance is the dominant elimination pathway, as was previously reported from animal studies [21]. While all patients in the trial had serum creatinine levels well below the exclusion criterion of 165 μmol/l, retrospective creatinine clearance calculations indicated that one patient who had a serum creatinine of 114 μmol/ fell in the moderate renal impairment category [39]. Although this issue did not specifically result in any AE or SAE in CELADEN, our current PK analysis suggests a greater awareness of renal function for future trials.

DENV cleared significantly more rapidly in patients with secondary dengue compared to primary dengue, confirming a previous finding [40]. Future trials of antiviral drugs for dengue drugs that use viremia as an endpoint may need to take this into account when calculating trial size in order to achieve adequate power. Although an ideal case Target Product Profile [41] for a dengue drug should be efficacy in both primary and secondary dengue, it is possible that for some early phase proof-of-concept studies, previous infection status could either be an inclusion criteria or a stratification variable to achieve balanced groups with respect to this parameter.

In the clinical management of dengue, platelet count is closely monitored for signs of incipient bleeding and progression to more serious DHF. Hemoconcentration is monitored through changes in hematocrit to decide whether to administer intravenous fluids and which type of fluid should be given [42]. For Proof of Concept and early stage human clinical trials, it is worth considering other parameters such as imaging leakage as an alternative early biomarker for clinical outcome in assessing potential drugs to treat dengue fever [43]. By gathering evidence to promote these biomarkers or hematology laboratory results to surrogate or valid clinical endpoints, it will become easier to evaluate other dengue drugs in the future.

The NGS analysis to obtain critical information on nucleotide changes did not reveal any suggestion that rapid development of resistance to celgosivir is an explanation for the low efficacy observed in our trial. However it does reinforce the need for a panel of strains that are representative for the various genotypes of DENV serotypes 1–4 to be examined as part of preclinical evaluation for efficacy, especially when the drug targets a host enzyme. Interestingly recent in vitro biochemical studies have confirmed that celgosivir’s mechanism of action is via inhibition of ER α-gulcosidases and also showed that the antiviral activity of celgosivir in primary human macrophages in vitro, inhibits DENV secretion with an EC50 of 5 μM [44]. These studies do lend support to celgosivir as a potential drug for detailed clinical analysis. Celgosivir’s efficacy on zika virus (ZIKV) has not been reported but given its close relationship with dengue virus within the flavivirus membership, it is possible that it may inhibit ZIKV using a similar mechanism. ZIKV, like DENV is spread by Aedes aegypti mosquitoes but its current association with widespread numbers of Guillain-Barre syndrome and microcephaly in babies born to mothers infected during the first trimester of pregnancy is unique and a cause of much concern [45].

Several lines of evidence suggest a trend towards a pharmacological effect of celgosivir in dengue patients. Celgosivir patients with secondary dengue had a slightly less decline in platelets. Only 1 of 13 (7.6%) of the secondary dengue patients treated with celgosivir had a platelet nadir below 20,000/μL compared to 2 of 5 (40%) in the placebo group. Secondary dengue patients treated with celgosivir also had a smaller difference between maximum and minimum hematocrit than secondary dengue patients in the placebo group. Other evidence of a trend towards pharmacologic activity include (i) the significantly lower level of TNFα in celgosivir-treated patients compared to placebo controls during the early stage of treatment, independent of prior infection status and (ii) enhanced NS1 clearance in secondary infection following celgosivir treatment [17].

In conclusion, lessons derived from the analysis of the secondary endpoint data of the CELADEN trial are a useful guide for future dengue drug trials. PK analyses and simulations show that alternate dosing regimens can achieve several-fold increases in steady-state trough concentrations with the same or modestly higher total dose. The difference in viremia kinetics for primary and secondary dengue underscores the need for balanced arms with respect to infection status in future trials. Pathological endpoints such as HCT and vascular leakage may be potentially better surrogate endpoints than viremia in assessing activity of dengue antivirals. We note that CELADEN was not powered to determine statistically significant differences in secondary endpoints. Such analyses are subject to inflated Type I error and merit cautious interpretation. However taken together, the trends in pharmcological activity are consistent with increasing celgosivir exposure. Since PK simulations of further divided doses suggest a potentially large increase in Cmin with a modest increase in AUC, which is supported by recent animal data [23], we plan to evaluate a revised dosing regimen of celgosivir in a Phase 2a to start in 2016 (NCT02569827).

## Supporting Information

S1 Fig Summary of inclusion and exclusion criteria for CELADEN study [17]. (DOCX)

S2 Fig Examination of intra-host genetic diversity by position for DENV1. The DENV1 genome was analyzed for positions having detectable, >1% and 5% non-consensus base calls for (A) placebo-treated and (B) celgosivir-treated samples. The number of strains with detectable (grey), >1% (black) and >5% (red) variance are plotted on the y-axis for each position in the DENV genome (x-axis). The letters indicate loci with a high degree of reproducibility (more than 25% of the samples).
(TIFF)

S3 Fig Examination of intra-host genetic diversity by position for DENV2. The DENV2 genome was analyzed for positions having detectable, >1% and 5% non-consensus base calls for (A) placebo-treated and (B) celgosivir-treated samples. The number of strains with detectable (grey), >1% (black) and >5% (red) variance are plotted on the y-axis for each position in the DENV genome (x-axis). The letters indicate loci with a high degree of reproducibility (more than 25% of the samples).
(TIFF)

S4 Fig Percentage of transitions and transversions observed in SNVs identified. For both (A) DENV1 and (B) DENV2, the majority (approximately 80% across all samples) of SNVs identified in our data set were transitions (A↔G, C↔T), and approximately 20% were transversions (A↔C, G↔T, G↔C, A↔T).
(TIFF)

S5 Fig Analysis of mutational coldspots for (A) DENV1 and (B) DENV2. Mutational coldspots were generated by grouping the samples based on treatment (Celgosivir (C) and Placebo (P)) and time point (tp). (A) For DENV1, more cold-spots were detected for celgosivir-treated samples (C-tp1 and C-tp2) than placebo samples (P-tp1 and P-tp2). (B) For DENV2, coldspots were detected mostly in NS3 and NS5 for placebo-treated samples, while coldspots were detected only in NS5 for celgosivir-treated samples.
(TIFF)

S1 Table Average SNVs/100 per gene of DENV genome at Time-point 3. (DOC)

S2 Table Selection pressures on the DENV genome, analyzed per gene at different time-points. (DOC)

S1 Text Celaden Clinical Protocol [17]. (DOC)
