# Supplementary material for: Extended Evaluation of Virological, Immunological and Pharmacokinetic Endpoints of CELADEN: A Randomized, Placebo-Controlled Trial of Celgosivir in Dengue Fever Patients
Source: PLoS Negl Trop Dis. 2016 Aug 10;10(8):e0004851. doi: 10.1371/journal.pntd.0004851 (PMC4980036; doi:10.1371/journal.pntd.0004851)
Supplement: S1 Fig — (DOCX) [file pntd.0004851.s001.docx]

**S1 Fig .** Summary of inclusion and exclusion criteria for CELADEN study.

**Inclusion criteria**

- Male or female, age 21-65 years.
- Fever of ≥ 38°C (directly measured or patient reported) of <48 hour duration.
- At least two of the following criteria indicating probable dengue infection: :Live or work in or recent travel to dengue endemic area; nausea or vomiting; presence of rash, aches or pains including headache retro-orbital, muscle or joint pain.
- Positive NS1 assay on point-of-care test kit or Panbio PCR.
- Able and willing to give written or oral informed consent.
- Willing to be an inpatient from study day 1 to 5, and to return to IMU on study days 7, 10 and 15.
- Willing to keep a diary of pain medication usage and side effects.

**Exclusion criteria**

- Clinical signs and symptoms for severe dengue, such as severe abdominal pain, persistent vomitting, clinical fluid accumulation, mucosal bleed, altered mental state, liver enlargement >2cm, systolic blood pressure <90mmHg, pulse pressure <20mmHg.
- Hematocrit >52% in males; > 46% in females.
- AST or ALT >1000U/L.
- Room air oxygen saturation <95%.
- Absolute neutrophil count <1500/µL.
- Platelet count <80,000/mm^3^.
- Creatinine >165 µmol/L in males; >130 µmol/L in females.
- Hemoglobin <13.0g/dL in males; <11.0g/dL in females.
- Total bilirubin >24 µmol/L.
- Serum creatinine kinase > 600U/L
- History of or presently active intestinal disorders such as peptic ulcers, intestinal ulcers, intestinal obstructions, intestinal hernias, ulcerative colitis, malabsorption disease, celiac disease, Roemheld’s syndrome (gastroesophageal regurgitation disease) or Crohn’s disease.
- Severe diarrhea (grade 2 or higher according to NIH clinical trial guidelines).
- Current usage of any anticoagulant drugs including, but not limited to, aspirin, warfarin, or clopidogrel.
- Any other clinically significant acute illness within 7 days prior to the first drug administration.
- History of severe drug and/or food allergies.
- Exposure to any new investigational agent within 30 days prior to the study drug administration.
- Clinically significant abnormal physical examination unrelated to dengue infection, chest X-ray or 12-lead electrocardiogram (ECG) at screening, such as QTc prolongation (>450msec).
- Females of childbearing potential who are pregnant, breastfeeding or unwilling to avoid pregnancy by the use of appropriate contraception, including oral and subcutaneous implantable hormonal contraceptives, condoms, diaphragm, or intrauterine device (IUD), during the peroid that the experimental drug is administered. Prospective female participants of childbearing potential must have a negative pregnancy test (point of care).
- Current significant medical conditions or illness including cardiac arrhythmia, cardiomyopathy or other cardiac disease, asthma, or other respiratory disease, diabetes mellitus, renal or hepatic impairment, thyroid disease, Parkinson’s disease, epilepsy or history of unexplained blackouts, immunocompromised state including known HIV infection, or any other illness that the Investigator considers should exclude the patient, especially those that require continuation of other medications likely to have an interaction with the study drug.
- Any condition that, in the opinion of the investigator, would complicate or compromise the study or well-being of the patient.
