# Supplementary figures and images for: Extended Evaluation of Virological, Immunological and Pharmacokinetic Endpoints of CELADEN: A Randomized, Placebo-Controlled Trial of Celgosivir in Dengue Fever Patients
Source: PLoS Negl Trop Dis. 2016 Aug 10;10(8):e0004851. doi: 10.1371/journal.pntd.0004851 (PMC4980036; doi:10.1371/journal.pntd.0004851)

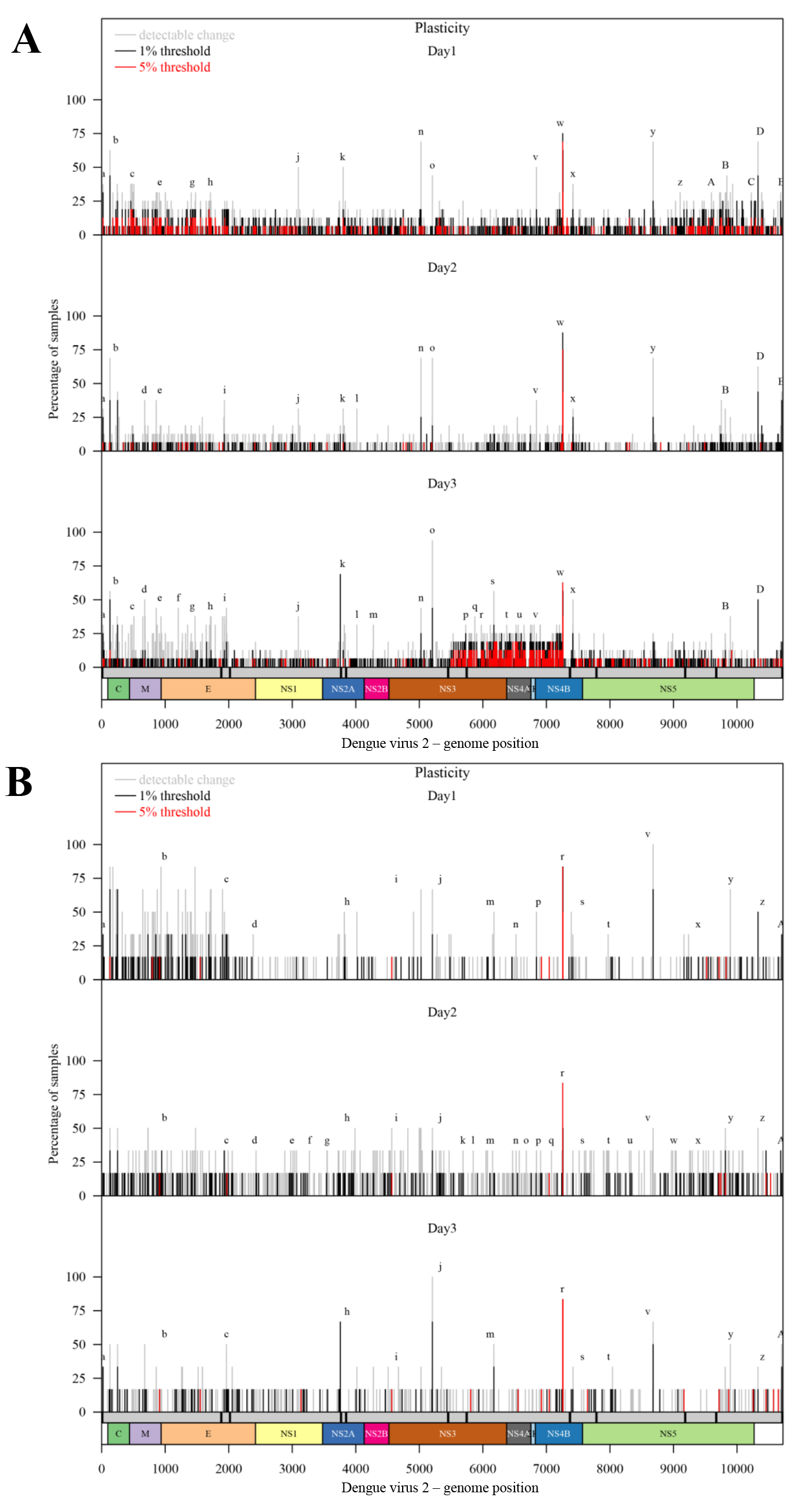

Supplement: S2 Fig — The DENV1 genome was analyzed for positions having detectable, >1% and 5% non-consensus base calls for (A) placebo-treated and (B) celgosivir-treated samples. The number of strains with detectable (grey), >1% (black) and >5% (red) variance are plotted on the y-axis for each position in the DENV genome (x-axis). The letters indicate loci with a high degree of reproducibility (more than 25% of the samples). (TIFF) [file pntd.0004851.s002.tiff]

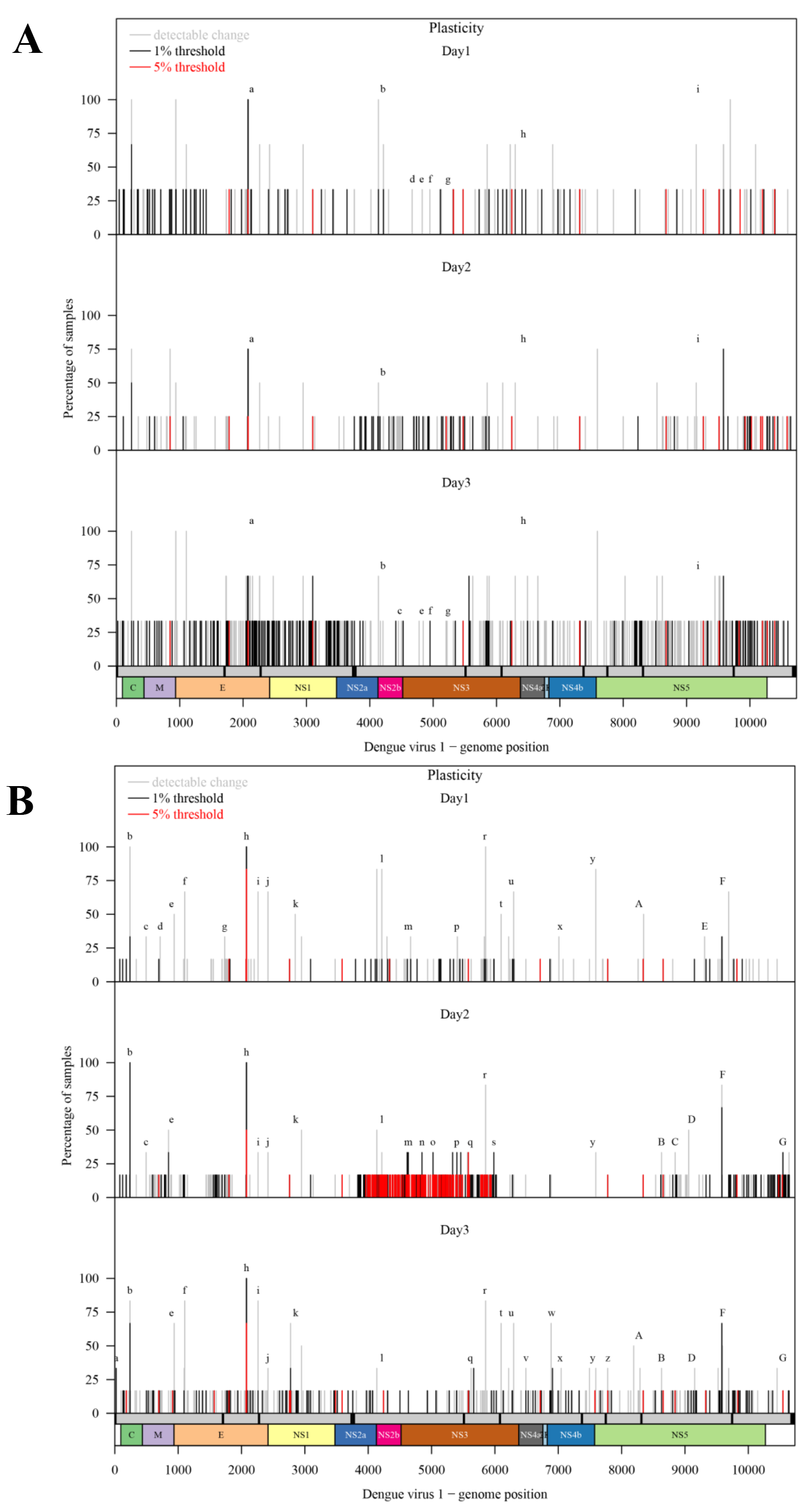

Supplement: S3 Fig — The DENV2 genome was analyzed for positions having detectable, >1% and 5% non-consensus base calls for (A) placebo-treated and (B) celgosivir-treated samples. The number of strains with detectable (grey), >1% (black) and >5% (red) variance are plotted on the y-axis for each position in the DENV genome (x-axis). The letters indicate loci with a high degree of reproducibility (more than 25% of the samples). (TIFF) [file pntd.0004851.s003.tiff]

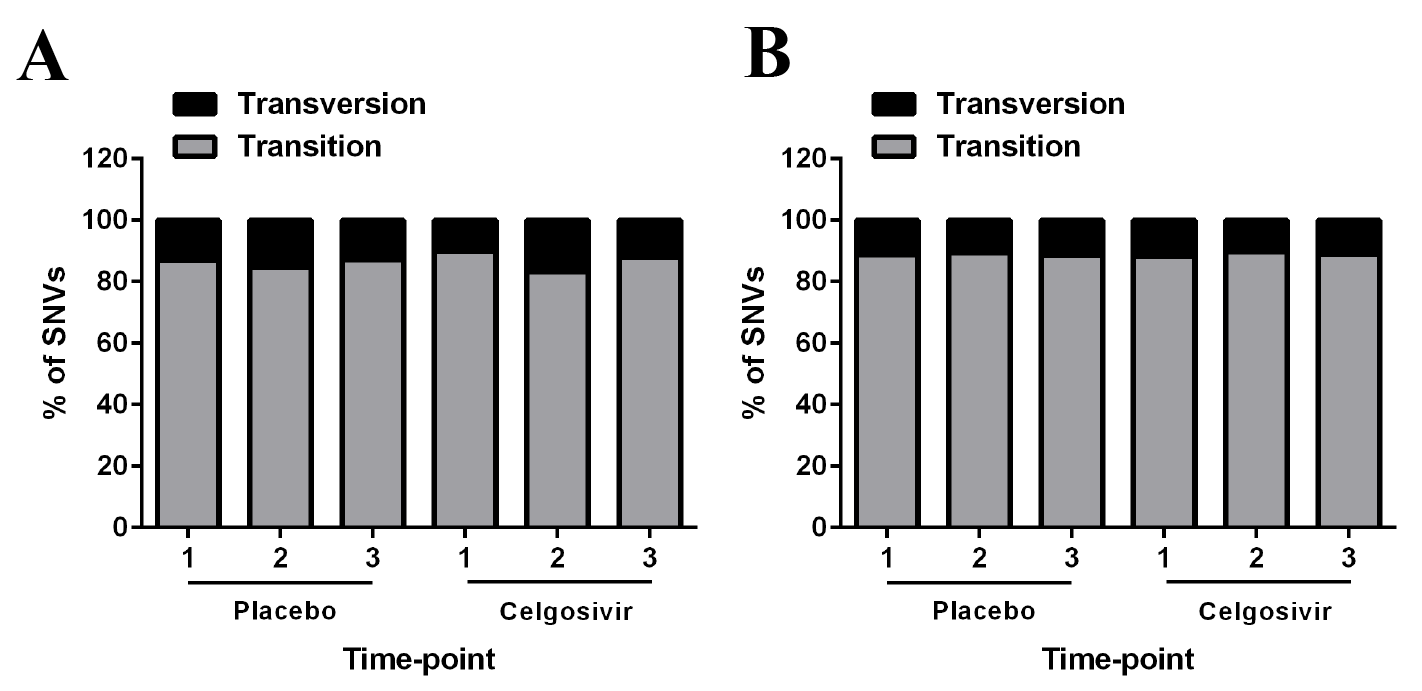

Supplement: S4 Fig — For both (A) DENV1 and (B) DENV2, the majority (approximately 80% across all samples) of SNVs identified in our data set were transitions (A↔G, C↔T), and approximately 20% were transversions (A↔C, G↔T, G↔C, A↔T). (TIFF) [file pntd.0004851.s004.tiff]

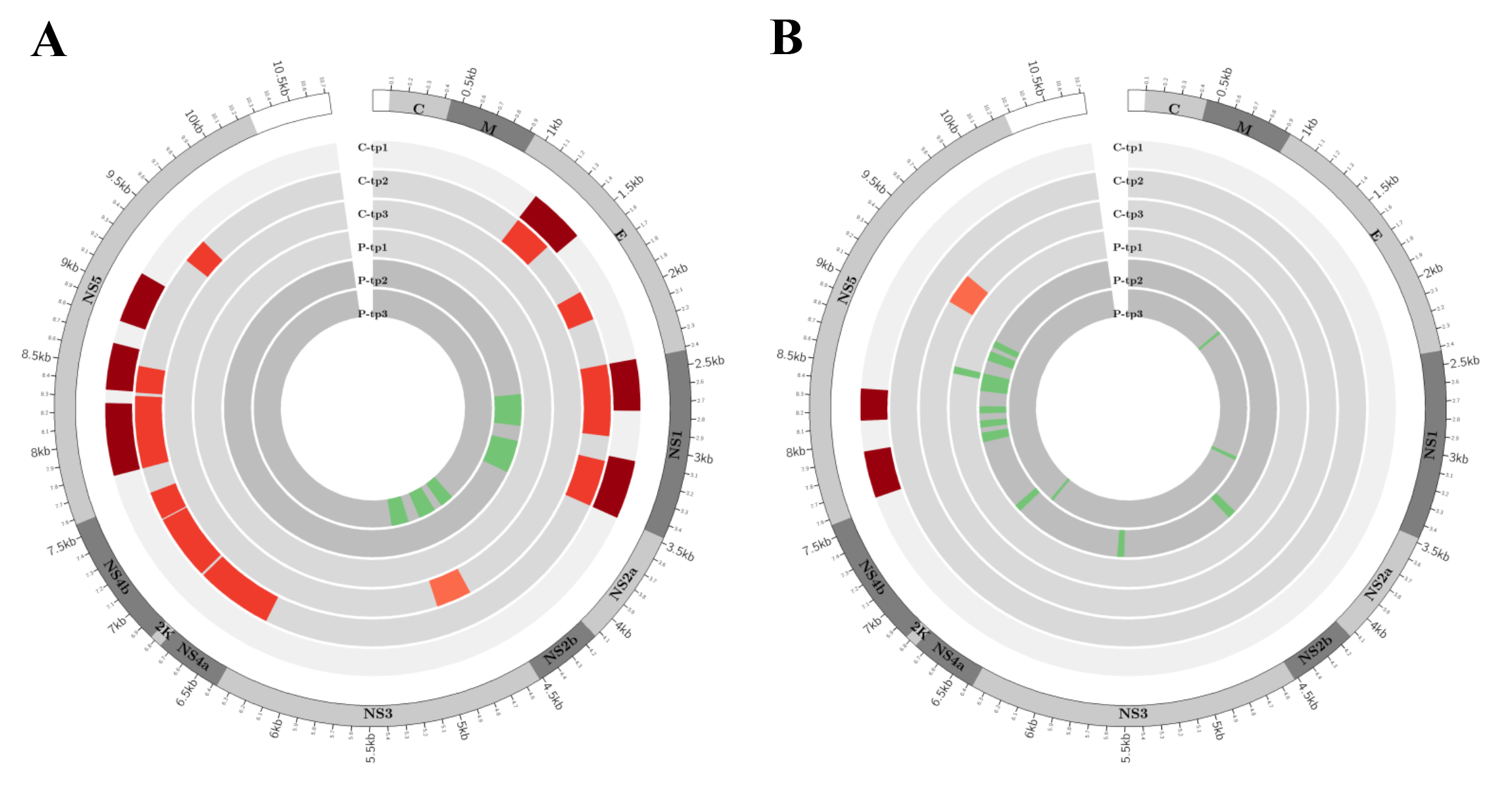

Supplement: S5 Fig — Mutational coldspots were generated by grouping the samples based on treatment (Celgosivir (C) and Placebo (P)) and time point (tp). (A) For DENV1, more cold-spots were detected for celgosivir-treated samples (C-tp1 and C-tp2) than placebo samples (P-tp1 and P-tp2). (B) For DENV2, coldspots were detected mostly in NS3 and NS5 for placebo-treated samples, while coldspots were detected only in NS5 for celgosivir-treated samples. (TIFF) [file pntd.0004851.s005.tiff]
