# Supplementary material for: Extended Evaluation of Virological, Immunological and Pharmacokinetic Endpoints of CELADEN: A Randomized, Placebo-Controlled Trial of Celgosivir in Dengue Fever Patients
Source: PLoS Negl Trop Dis. 2016 Aug 10;10(8):e0004851. doi: 10.1371/journal.pntd.0004851 (PMC4980036; doi:10.1371/journal.pntd.0004851)
Supplement: S1 Table — (DOC) [file pntd.0004851.s006.doc]

**S1 Table. Average SNVs/100 per gene of DENV genome at Time-point 3**

|  |  | **SNVs/100** |  |  |
| --- | --- | --- | --- | --- |
|  | **Placebo (DENV1)** | **Celgosivir (DENV1)** | **Placebo (DENV2)** | **Celgosivir (DENV2)** |
| **Polyprotein** | 1.29 | 0.61 | 1.38 | 0.66 |
| **C** | 0.81 | 0.74 | 1.80 | 0.93 |
| **M** | 0.76 | 0.71 | 1.92 | 0.91 |
| **E** | 2.09 | 0.97 | 1.33 | 1.01 |
| **NS1** | 1.99 | 0.59 | 0.94 | 0.60 |
| **NS2A** | 1.11 | 0.51 | 0.96 | 0.64 |
| **NS2B** | 0.45 | 0.26 | 0.84 | 0.56 |
| **NS3** | 0.67 | 0.51 | 1.58 | 0.61 |
| **NS4A** | 0.73 | 0.48 | 2.97 | 0.53 |
| **2K** | 1.49 | 0.25 | 2.24 | 0.50 |
| **NS4B** | 0.70 | 0.56 | 2.03 | 0.81 |
| **NS5** | 1.58 | 0.59 | 1.06 | 0.43 |
