# Supplementary material for: Extended Evaluation of Virological, Immunological and Pharmacokinetic Endpoints of CELADEN: A Randomized, Placebo-Controlled Trial of Celgosivir in Dengue Fever Patients
Source: PLoS Negl Trop Dis. 2016 Aug 10;10(8):e0004851. doi: 10.1371/journal.pntd.0004851 (PMC4980036; doi:10.1371/journal.pntd.0004851)
Supplement: S2 Table — (DOC) [file pntd.0004851.s007.doc]

**S2 Table. Selection pressures on the DENV genome, analyzed per gene at different time-points**

| **DENV1 Time point 1** | | | | | |
| --- | --- | --- | --- | --- | --- |
| **Gene** | **P (NS)** | **P (S)** | **C (NS)** | **C (S)** | **p-value (Fisher's exact test)** |
| **C** | 0 | 16 | 0 | 9 | 1.00 |
| **M** | 0 | 11 | 0 | 5 | 1.00 |
| **E** | 0 | 39 | 3 | 50 | 0.26 |
| **NS1** | 0 | 14 | 0 | 7 | 1.00 |
| **NS2A** | 0 | 8 | 0 | 18 | 1.00 |
| **NS2B** | 0 | 7 | 1 | 16 | 1.00 |
| **NS3** | 0 | 30 | 1 | 61 | 1.00 |
| **NS4A** | 0 | 4 | 0 | 2 | 1.00 |
| **2K** | 0 | 0 | 0 | 0 | 1.00 |
| **NS4B** | 0 | 12 | 0 | 7 | 1.00 |
| **NS5** | 0 | 37 | 0 | 42 | 1.00 |

| **DENV1 Time point 2** | | | | | |
| --- | --- | --- | --- | --- | --- |
| **Gene** | **P (NS)** | **P (S)** | **C (NS)** | **C (S)** | **p-value (Fisher's exact test)** |
| **C** | 0 | 6 | 2 | 9 | 0.51 |
| **M** | 3 | 8 | 1 | 27 | 0.06 |
| **E** | 0 | 26 | 4 | 58 | 0.31 |
| **NS1** | 0 | 5 | 0 | 6 | 1.00 |
| **NS2A** | 1 | 17 | 3 | 48 | 1.00 |
| **NS2B** | 0 | 22 | 2 | 53 | 1.00 |
| **NS3** | 1 | 50 | 5 | 187 | 1.00 |
| **NS4A** | 0 | 1 | 0 | 1 | 1.00 |
| **2K** | 0 | 0 | 0 | 0 | 1.00 |
| **NS4B** | 0 | 4 | 0 | 2 | 1.00 |
| **NS5** | 0 | 48 | 0 | 71 | 1.00 |

| **DENV1 Time point 3** | | | | | |
| --- | --- | --- | --- | --- | --- |
| **Gene** | **P (NS)** | **P (S)** | **C (NS)** | **C (S)** | **p-value (Fisher's exact test)** |
| **C** | 0 | 11 | 0 | 15 | 1.00 |
| **M** | 0 | 15 | 0 | 21 | 1.00 |
| **E** | 4 | 120 | 3 | 83 | 1.00 |
| **NS1** | 4 | 80 | 1 | 36 | 1.00 |
| **NS2A** | 1 | 28 | 1 | 19 | 1.00 |
| **NS2B** | 0 | 7 | 0 | 6 | 1.00 |
| **NS3** | 1 | 49 | 0 | 57 | 0.47 |
| **NS4A** | 0 | 11 | 0 | 11 | 1.00 |
| **2K** | 0 | 4 | 0 | 1 | 1.00 |
| **NS4B** | 0 | 21 | 0 | 25 | 1.00 |
| **NS5** | 5 | 165 | 1 | 95 | 0.42 |

| **DENV2 Time point 1** | | | | | |
| --- | --- | --- | --- | --- | --- |
| **Gene** | **P (NS)** | **P (S)** | **C (NS)** | **C (S)** | **p-value (Fisher's exact test)** |
| **C** | 4 | 74 | 4 | 35 | 0.44 |
| **M** | 7 | 132 | 6 | 59 | 0.36 |
| **E** | 11 | 294 | 8 | 127 | 0.31 |
| **NS1** | 5 | 184 | 0 | 22 | 1.00 |
| **NS2A** | 8 | 102 | 2 | 21 | 0.68 |
| **NS2B** | 0 | 65 | 0 | 8 | 1.00 |
| **NS3** | 6 | 280 | 0 | 61 | 0.60 |
| **NS4A** | 1 | 70 | 0 | 9 | 1.00 |
| **2K** | 0 | 11 | 0 | 0 | 1.00 |
| **NS4B** | 10 | 137 | 2 | 29 | 1.00 |
| **NS5** | 27 | 511 | 1 | 71 | 0.23 |

| **DENV2 Time point 2** | | | | | |
| --- | --- | --- | --- | --- | --- |
| **Gene** | **P (NS)** | **P (S)** | **C (NS)** | **C (S)** | **p-value (Fisher's exact test)** |
| **C** | 3 | 52 | 0 | 24 | 0.55 |
| **M** | 8 | 87 | 4 | 55 | 1.00 |
| **E** | 8 | 221 | 6 | 116 | 0.57 |
| **NS1** | 2 | 114 | 1 | 68 | 1.00 |
| **NS2A** | 4 | 65 | 4 | 45 | 0.72 |
| **NS2B** | 0 | 22 | 0 | 35 | 1.00 |
| **NS3** | 2 | 159 | 2 | 141 | 1.00 |
| **NS4A** | 1 | 50 | 1 | 35 | 1.00 |
| **2K** | 0 | 6 | 0 | 3 | 1.00 |
| **NS4B** | 13 | 114 | 3 | 52 | 0.40 |
| **NS5** | 9 | 231 | 10 | 185 | 0.49 |

| **DENV2 Time point 3** | | | | | |
| --- | --- | --- | --- | --- | --- |
| **Gene** | **P (NS)** | **P (S)** | **C (NS)** | **C (S)** | **p-value (Fisher's exact test)** |
| **C** | 3 | 52 | 0 | 24 | 0.55 |
| **M** | 8 | 87 | 4 | 55 | 1.00 |
| **E** | 8 | 221 | 6 | 116 | 0.57 |
| **NS1** | 2 | 114 | 1 | 68 | 1.00 |
| **NS2A** | 4 | 65 | 4 | 45 | 0.72 |
| **NS2B** | 0 | 22 | 0 | 35 | 1.00 |
| **NS3** | 2 | 159 | 2 | 141 | 1.00 |
| **NS4A** | 1 | 50 | 1 | 35 | 1.00 |
| **2K** | 0 | 6 | 0 | 3 | 1.00 |
| **NS4B** | 13 | 114 | 3 | 52 | 0.40 |
| **NS5** | 9 | 231 | 10 | 185 | 0.49 |

P (NS): Placebo (Non-synonymous); P (S): Placebo (Synonymous); C (NS): Celgosivir (Non-synonymous); C (S): Celgosivir (Synonymous)
