# Supplementary material for: Extended Evaluation of Virological, Immunological and Pharmacokinetic Endpoints of CELADEN: A Randomized, Placebo-Controlled Trial of Celgosivir in Dengue Fever Patients
Source: PLoS Negl Trop Dis. 2016 Aug 10;10(8):e0004851. doi: 10.1371/journal.pntd.0004851 (PMC4980036; doi:10.1371/journal.pntd.0004851)
Supplement: S1 Text — (DOC) [file pntd.0004851.s008.doc]

See also Ref 16

Clinical Protocol EID-DF-01

V 2.14

08 Mar 2013

**Title:**

**Cel**gosivir Proof of Concept Trial for Treatment of **A**cute **Den**gue Fever (**CELADEN**)

**Subtitle:**

Randomized, Double-Blind, Placebo-Controlled, Phase 1b Clinical Study to Evaluate the Activity, Pharmacokinetics, Safety and Tolerability of Celgosivir in Adults with Confirmed Dengue Fever

**Principal Investigator:**

LOW Jenny Guek Hong, MBBS, MRCP (UK), MPH

Infectious Diseases Consultant

Singapore General Hospital

| **Study Sponsor** | **Study Monitor** |
| --- | --- |
| Singapore General Hospital (SGH)  Block 6, Level 7  Singapore 169608 | Singapore Clinical Research Institute  31 Biopolis Way Nanos #02-01  Singapore 138669 |

| **Protocol Author** | **Protocol Reviewers** |
| --- | --- |
| Cynthia Sung, PhD, Adjunct Asst. Professor  Duke-NUS Graduate Medical School  Program for Emerging Infectious Diseases  8 College Rd., Floor 9  Singapore 169857 | Subhash Vasuvedan, PhD, Assoc. Professor Eng Eong Ooi, BM, BS, PhD, Assoc. Prof  Duke-NUS Graduate Medical School  Jenny G. Low, MBBS, MRCP (UK), MPH  Infectious Diseases Consultant, SGH |

Confidential and Proprietary

This document contains proprietary and confidential information of the Duke-NUS Graduate Medical School. Acceptance of this document constitutes agreement by the recipient that no unpublished information contained herein will be published or disclosed without prior written approval from Duke-NUS.

**TABLE OF CONTENTS**

1 Background [2](#__RefHeading___Toc202434857)

2 Objectives and hypotheses [4](#__RefHeading___Toc202434858)

2.1 Primary objectives [4](#__RefHeading___Toc202434859)

2.2 Secondary objectives [5](#__RefHeading___Toc202434860)

2.3 Exploratory objectives [5](#__RefHeading___Toc202434861)

3 Study Design [6](#__RefHeading___Toc202434862)

3.1 Summary [6](#__RefHeading___Toc202434863)

3.2 Selection and Withdrawal of Patients [7](#__RefHeading___Toc202434864)

3.2.1 Inclusion Criteria [7](#__RefHeading___Toc202434865)

3.2.2 Exclusion Criteria [8](#__RefHeading___Toc202434866)

3.2.3 Premature Withdrawal of Patients from Study [9](#__RefHeading___Toc202434867)

4 Study Medication [10](#__RefHeading___Toc202434868)

4.1 Drug Name, Formulation and Storage [10](#__RefHeading___Toc202434869)

4.2 Dosing Regimen [10](#__RefHeading___Toc202434870)

4.3 Diet [10](#__RefHeading___Toc202434871)

4.4 Concomitant Medications [10](#__RefHeading___Toc202434872)

4.5 Drug Accountability [11](#__RefHeading___Toc202434873)

5 Study Procedures [11](#__RefHeading___Toc202434874)

5.1 Methods and Assessments [11](#__RefHeading___Toc202434875)

5.2 Randomization [12](#__RefHeading___Toc202434876)

5.3 Blinding [12](#__RefHeading___Toc202434877)

5.4 Data Safety Monitoring [12](#__RefHeading___Toc202434878)

5.5 Data Management [13](#__RefHeading___Toc202434879)

5.6 Criteria for Discontinuation of Patient Study Drug and for Study Termination [13](#__RefHeading___Toc202434880)

6 Statistical Considerations [16](#__RefHeading___Toc202434881)

6.1 Sample size calculation [16](#__RefHeading___Toc202434882)

6.2 Statistical analysis [16](#__RefHeading___Toc202434883)

7 Safety Assessment [18](#__RefHeading___Toc202434884)

7.1 Definitions [18](#__RefHeading___Toc202434885)

7.2 Adverse Event Grading, Causality, and Expectedness [18](#__RefHeading___Toc202434886)

7.3 Serious Adverse Event Reporting [19](#__RefHeading___Toc202434887)

8 Study Monitoring [19](#__RefHeading___Toc202434888)

9 References [20](#__RefHeading___Toc202434889)

# Background

Globally, dengue infections result in more than 20,000 deaths, nearly 500,000 hospitalized cases and anywhere between 50-100 million human infections annually [WHO 2009]. Dengue infection is caused by one of four immunologically distinct serotypes of the dengue virus (DENV 1-4). The virus is spread by the urban-breeding mosquito *Aedes aegypti*. Usually, infection with any one of the four DENV serotypes leads to mild, self-limiting dengue fever with lifelong immunity to that specific serotype. Epidemiological evidence also indicates that 90% of the severe and potentially fatal dengue diseases, dengue hemorrhagic fever (DHF) or dengue shock syndrome (DSS) occur during secondary heterotypic infections where the protective antibody from a prior infection takes on a pathogenic role, so-called Antibody Dependent Enhancement (ADE) [Fink 2006, Halstead 2007]. The antibody response triggers a systemic inflammatory reaction resulting in vascular leakage. There is no approved preventative vaccine or antiviral treatment for dengue disease. The World Health Organization has listed dengue fever as an emerging and uncontrolled disease [Remme 2002]. In Singapore, despite expenditures by the National Environment Agency of SG$63 million per year on mosquito vector control, the average number of dengue cases from 2007 to 2010 is >6000 per year. Hence there is a need for an antiviral compound in Singapore, and even more so in other tropical countries that do not have as effective vector control as Singapore.

Celgosivir is a butyl ester derivative of castanospermine, a natural product derived from the seeds of *Castanospermum australe*. It exerts antiviral activity by inhibiting host alpha-glucosidases I and II, enzymes essential for proper folding of dengue-encoded proteins such as E, prM and NS1 [Courageot 2000, Rathore 2011]. Fluorescence microscopy images demonstrate that celgosivir inhibits production of dengue NS1 protein in infected cells [Rathore 2011]. Without glycosylated proteins, infectious viral particles accumulate in the endoplasmic reticulum (ER), and mature virions cannot be produced. Since the drug target is a host enzyme required for viral maturation, the potential for development of resistance is expected to be lower than a drug directed against a viral enzyme. Celgosivir exhibits activity against a variety of laboratory and clinical dengue strains of DENV1-4, with submicromolar EC50 values. Celgosivir has been tested in AG129 mice lacking interferon- α, β and γ receptors, which were infected with a clinical strain of DENV-2. In this model, celgosivir significantly reduced viremia by 88% and 55%, respectively, when given at the time of infection and when treatment was delayed by a day [Schul 2007]. In a lethal model of viremia, mice were pretreated with an antibody against DENV E protein to model ADE and then infected with a mouse-adapted strain of DENV-2. In this animal model, untreated mice had 0% survival by Day 5, whereas mice treated with 50 mg/kg celgosivir PO bid, 100% were still alive at Day 12 (p = 0.0001). Even when treatment was delayed by 24 and 48 hr, celgosivir was still able to confer protection, with 75% and 50% survival, respectively to Day 12. Treatment at 10, 25, and 50 mg/kg PO bid results in 12%, 62% and 100% survival. When mice are treated once daily at 100 mg/kg PO, no mice survive past day 6 compared to 100% survival at 50 mg/kg twice daily, indicating that the drug, when divided into two doses, is more effective that giving the same total dose once a day.

Celgosivir has been evaluated in humans as a potential treatment against HIV and Hepatitis C (HCV). Marion Merrell Dow, which subsequently became Hoescht Marion Roussel, conducted the HIV program in the US and Europe in the 1990s. The US program had regulatory oversight under FDA IND 44,158, filed by Marion Merrell Dow in 1994. An IND is only granted for first-in-human studies when nonclinical toxicology performed under GLP standards indicates that the molecule has an adequate safety profile for human testing. The studies included acute studies in mice, rats and dogs, as well as repeat dose studies in rats, dogs and nonhuman primates, for up to 30 days. The main clinical and laboratory findings in a 28-day rat study were decreased body weight and food consumption, increased liver enzymes AST and ALT, and decreased platelets (only at 100 mg/kg/day). Pathological findings were intralysosomal glycogen accumulation and degeneration of hair follicles that were reversible upon discontinuation of drug. The NOAEL was <10 mg/kg/day. Dogs had a similar spectrum of toxicology findings, and in addition, experienced emesis and diarrhea in a 30-day study. The NOAEL in dogs was 5 mg/kg/day in females and between 1 and 5 mg/kg/day in males. Acute cardiovascular and CNS safety pharmacology studies had no significant findings. Genotoxicity tests (Ames, forward mutation assay, and chromosomal abberation assays) were all negative. In the early 2000s, Migenix pursued celgosivir as a treatment for HCV (another member of the *Flaviviridae* family) under regulatory oversight by Health Canada.

The human safety and tolerability of celgosivir has been tested in over 600 subjects at doses between 10 and 600 mg [Kaita 2007]. The drug has been chronically dosed with daily dosing for 12 weeks in over 340 HIV patients and 65 HCV patients. It also has been evaluated in some HCV patients for between 24 and 48 weeks at 400 mg PO daily [Durantel 2009]. Celgosivir is well absorbed with a Tmax <1 h and a half-life of 13-30 hr, with dose proportional exposure between 10 and 450 mg [Stoltz 1996, Sorbera 2005]. Celgosivir is rapidly converted to castanospermine, which is excreted unchanged in the urine, with no other metabolites detected. Adverse events were largely gastrointestinal, namely flatulence and diarrhea, of mild to moderate intensity. Asymptomatic elevations in serum creatine kinase were also observed, which were reversed within 2 weeks after discontinuation of the drug. No serious adverse events have been reported. Although celgosivir exhibited antiviral activity, it was not superior to existing treatments for HIV and HCV, and further development for those diseases was discontinued.

Celgosivir is in the class of alpha-glucosidase inhibitors. Another alpha-glucosidase I inhibitor, acarbose, is approved in the US, Europe and Singapore for treatment of Type 2 diabetes mellitus. The recommended dose of acarbose is 50 to 100 mg TID for a total daily dose of 150 to 300 mg.

In summary, the activity of celgosivir in several nonclinical *in vitro* dengue assays, reduction of viremia and significantly improved survival in animal models of dengue infection after celgosivir treatment, a record of safety and tolerability in human clinical trials for HIV and HCV up to 400 mg QD for 12 weeks, and the urgent need for dengue antiviral agent provide a compelling rationale for undertaking a proof-of-concept clinical trial of celgosivir for treatment of acute dengue fever.

Initiation of the trial will be contingent on obtaining approval of the relevant Investigational Review Board and the Health Sciences Authority of Singapore.

# Objectives and hypotheses

## Primary objectives

To investigate the effectiveness of celgosivir for treating acute dengue fever in adult patients

1. Hypothesis: The mean of virological log reduction (VLR) at Day 2, Day 3 and Day 4 will be greater for patients taking celgosivir than for those taking placebo (virological endpoint)
2. Hypothesis: The AUC above baseline of the fever curve for patients taking celgosivir will be lower than for those taking placebo (clinical endpoint)

## Secondary objectives

1. To investigate the safety and tolerability of celgosivir

Hypothesis: The proportion of patients experiencing adverse events (AEs) or serious adverse events (SAEs) will be the same or lower in patients taking celgosivir than those taking placebo.

1. To investigate the effect of celgosivir on the time to viral clearance of dengue fever patients

Hypothesis: The time of viral clearance will be shorter for patients taking celgosivir than for those taking placebo

1. To investigate the effect of celgosivir on the serum NS1 of dengue fever patients

Hypotheses:

1. The mean serum NS1 concentration between Day 2 and Day 5 inclusive will be lower for patients taking celgosivir than for those taking placebo after adjusting for primary and secondary infection
2. The time to NS1 clearance will be shorter for patients taking celgosivir than for those taking placebo
3. To investigate the effect of celgosivir on the change in leukocyte and platelet counts of dengue fever patients

Hypothesis: The maximal change from baseline in leukocyte and platelet counts from Day 2 to Day 5 will be lower in patients taking celgosivir than those taking placebo

1. To investigate the effect of celgosivir on hemoconcentration in dengue fever patients

Hypothesis: The mean maximal hemoconcentration from Day 2 to Day 5 will be lower in patients taking celgosivir than those taking placebo

1. To characterize the population pharmacokinetics (PK) of celgosivir

Hypothesis: The clearance and volume of distribution of celgosivir will be dependent on weight and independent of age and sex.

## Exploratory objectives

1. To investigate the effect of celgosivir on the immunological profile of dengue fever patients

Hypothesis: The maximal change from baseline between Day 2 and 5 will be lower for patients taking celgosivir than for those taking placebo for the following cytokines: (a) TNF-α, (b) IL-6, (c) IL-12, (d) interferon-γ, (e) IP-10 and (f) MCP-1.

1. To investigate the effect of celgosivir on the use of analgesic medications of dengue fever patients

Hypothesis: Patients taking celgosivir will use less analgesic medications from Day 1 to Day 5 than those taking placebo.

1. To investigate the effect of celgosivir on the intensity of joint and muscle pain of dengue fever patients

Hypothesis: Patients taking celgosivir will experience a lower intensity of joint and muscle pain from Day 2 to Day 5 than those taking placebo.

# Study Design

## Summary

This is a randomized, double blind, placebo-controlled, Phase 1b clinical study of celgosivir in adults age 21 to 60 with fever ≥38°C of ≤48 hr duration, who meet at least two criteria indicating probable dengue infection, and who test positive for dengue infection by a point of care NS1 strip assay. Patients meeting all inclusion and exclusion criteria will be enrolled and admitted to the Investigational Medicine Unit (IMU).

Table 1 Study Design

| Cohort | N | Dose | Key Inclusion Criteria | Primary Outcome | Secondary Outcomes | Exploratory Outcomes |
| --- | --- | --- | --- | --- | --- | --- |
| 1 | 25 | Placebo | • Fever ≥38°C of ≤48 hr duration | • Viremia (laboratory) | • Safety & tolerability & PK |  |
|  |  |  |  |  | • Time to viral clearance | • Immuno-profiling |
|  |  |  | • positive NS1 strip assay |  | • NS1 | • Usage of analgesics |
| 2 | 25 | 400 mg loading dose then 200 mg q12hr | • 2 symptoms of probable dengue infection | • Fever (clinical) | • Leukocyte & Platelet count, Hemocon-centration | • Duration & magnitude of symptoms |
|  |  |  |  |  |  |  |

Fifty (50) patients will be randomized 1:1 to celgosivir or placebo. Capsules of placebo or celgosivir will be administered for 5 days. Patients will be given an initial loading dose of 400 mg followed by a maintenance dose of 200 mg every 12 hr for a total of nine (9) doses. Drug should be taken 1 hr before consuming food or 2 hr afterwards. Patients will be placed on a special diet of protein, vitamins, and glucose containing minimal complex sugars or starches. Oral intake of fluids will be recorded. While in hospital from Day 1 to Day 5, daily clinical exams will be conducted, and blood samples will be collected for plasma viral load, NS1, hematology, clinical chemistry, cytokine levels and safety observations. During this period, eight serum samples will be obtained for drug levels. On Study Day 5, after blood draws, administration of the last dose, safety assessments, determination of risk for DHF or DSS, and satisfactory clinical status, the patient will be discharged. Patients at risk for or who have dengue hemorrhagic fever (DHF) or dengue shock syndrome (DSS) will remain in hospital until fit for discharge. Patients who progress to DHF or DSS may require intravenous fluids, platelet and/or blood transfusions and evaluations from other clinical specialists. At discharge, the patient will be asked to return on Study Days 7, 10, and 15 for blood sampling and safety assessments. Patients will complete visual analog scales for joint and muscle pain (VASP), and be evaluated for safety and tolerability daily while in hospital and at each subsequent outpatient visit. While in hospital, concomitant usage of analgesics will be recorded. As an outpatient, patients will keep a diary of concomitant usage of analgesics. If the patient remains in hospital past day 5, planned outpatient study assessments will be collected in hospital.

The patient will complete the study on Study Day 15 after the investigator has completed the final assessment, unless ongoing adverse events require monitoring. For subjects prematurely terminating participation in the study, assessments planned for Study Day 15 will be obtained if possible, and study completion evaluations must be performed.

Serum or plasma samples for viremia, quantitative NS1, immunological profile, DNA and RNA analysis, and PK will be transferred to Duke-NUS, Singapore Immunology Network, National University of Singapore or other laboratories for analysis and/or storage. The period of storage will not exceed 5 years.

After all study assessments have been completed on all patients and the database is locked, unblinding will occur. The primary virological endpoint will be the mean of virological log reduction (VLR) Study Day 2 to 4 between placebo and study drug groups. The primary clinical endpoint will be a reduction in fever AUC above baseline. Secondary endpoints for antiviral activity will be time to viral clearance, serum NS1 levels, leukocyte and platelet count, and hemoconcentration.

Secondary endpoints for assessing safety and tolerability are the number and severity of all adverse events and severe adverse events related to study drug compared to placebo using the 2009 WHO classification for grading adverse drug events. In addition, eight (8) samples for celgosivir serum concentrations will be obtained for population PK analysis.

Exploratory endpoints will be concentrations of various cytokines between placebo and treated groups, and changes in RNA expression of immunomodulatory genes. The trial will also assess the validity of the VASP and concomitant usage of analgesics between placebo and treatment groups as other measures of drug activity.

## Selection and Withdrawal of Patients

### Inclusion Criteria

1. Male or female, age 21-65 years
2. Fever of ≥ 38°C (directly measured or patient reported) of ≤ 48 hr duration.
3. At least two of the following criteria indicating probable dengue infection:
   1. Live or work in or recent travel to dengue endemic area
   2. Nausea and vomiting
   3. Presence of rash
   4. Aches and pains, including headache, or retro-orbital, muscle or joint pain
4. Positive NS1 strip assay or PCR
5. Able and willing to give written or oral informed consent
6. Willing to be an inpatient for from Study Day 1 to 5, and to return to the IMU on Study Days 7, 10, and 15.
7. Willing to keep a diary of pain medication usage and side effects

### Exclusion Criteria

1. Clinical signs and symptoms for severe dengue, such as:
   1. Severe abdominal pain
   2. Persistent vomiting
   3. Clinical fluid accumulation
   4. Mucosal bleed
   5. Altered mental state
   6. Liver enlargement > 2 cm
   7. Systolic blood pressure < 90 mm Hg
   8. Pulse pressure < 20 mm Hg
2. A person with any of the following laboratory values:
3. Hematocrit >52% males; >46% females
4. AST or ALT ≥ 1000 U/L
5. Room air oxygen saturation <95%
6. Absolute neutrophil count <1500 /µL
7. Platelet count <80,000/mm^3^
8. Creatinine >165 µmol/L males; > 130µmol/L females
9. Hemoglobin <13.0 g/dL males; <11.0 g/dL females
10. Total Bilirubin > 24 µmol/L
11. Serum creatine kinase > 600 U/L
12. History of or presently active intestinal disorders such as peptic ulcers, intestinal ulcers, intestinal obstructions, intestinal hernias, ulcerative colitis, malabsorption syndrome, celiac disease, Roemheld’s syndrome (gastroesophageal regurgitation disease) or Crohn’s disease
13. Severe diarrhea (grade 2 or higher according to NIH clinical trials guidelines)
14. Current usage of any anticoagulant drugs including, but not limited to, aspirin, warfarin, or clopidogrel
15. Any other clinically significant acute illness within 7 days prior to first study drug administration.
16. History of severe drug and/or food allergies.
17. Exposure to any new investigational agent within 30 days prior to the study drug administration.
18. Clinically significant abnormal physical examination unrelated to dengue infection, chest X-ray or 12-lead electrocardiogram (ECG) at screening, such as QTc prolongation (>450 msec).
19. Females of childbearing potential who are pregnant, breast feeding, or unwilling to avoid pregnancy by the use of appropriate contraception, including oral and subcutaneous implantable hormonal contraceptives, condoms, diaphragm, or intrauterine device (IUD), during the period that the experimental drug is administered. Prospective female participants of childbearing potential must have a negative pregnancy test (point of care)
20. Current significant medical condition or illness including cardiac arrhythmias, cardiomyopathy or other cardiac disease, asthma or other respiratory disease, diabetes mellitus, renal or hepatic impairment, thyroid disease, Parkinson’s disease, epilepsy or history of unexplained blackouts, immunocompromised state including known HIV infection, or any other illness that the Investigator considers should exclude the patient, especially those that require continuation of other medications likely to have an interaction with the study drug.
21. Any condition that would render the informed consent invalid, or limit the ability of the patient to comply with the study requirements.
22. Any condition that, in the opinion of the investigator, would complicate or compromise the study or well being of the patient.

### Premature Withdrawal of Patients from Study

Patients are free to withdraw consent and discontinue participation at any time without penalty and are not obliged to give a reason. Patients may be withdrawn/removed, if necessary, to protect their health or the integrity of the study. The Investigator also has the right to withdraw patients from the study in the event of intercurrent illness, adverse events, treatment failures, protocol violations, administrative, or other reasons. When a patient withdraws from the study, all safety data normally required at the completion of the study will be obtained, where possible. All details available will be reported and recorded for any patient that withdraws or is removed from the study.

If the reason for removal of a patient from the study is an adverse event, intercurrent illness, or an abnormal laboratory value, the principal specific event or test will also be recorded on the case report form (CRF).

CRFs are required for all patients who receive study medication, even if they subsequently withdraw from the study before all assessments are completed. All sections of the CRF, up to the time of withdrawal, are to be completed. Efforts should be made to obtain complete information regarding the tolerability and safety of the test drug. This means that asking for and recording Adverse Events should continue whenever possible. Patients who withdraw from the trial will not be replaced.

All patients who withdraw early from the study but have received all days of study medication will be included in the analysis of effectiveness.

All patients who withdraw from the study but receive at least one dose of study medication will be included in the analysis of safety.

# Study Medication

## Drug Name, Formulation and Storage

Celgosivir is a butyl ester derivative of castanospermine, also known as 6-butanoyl castanospermine, castanospermine 6-butryate in the hydrochloride salt form or Bu-Cast. Its IUPAC name is [(1S,6S,7S,8R,8aR)-1,7,8-trihydroxy-1,2,3,5,6,7,8,8a-octahydroindolizin-6-yl] butanoate hydrochloride. The chemical formula is C_12_H_21_NO_5_•HCl, molecular weight 295.75. The chemical structure is shown in Figure 1.


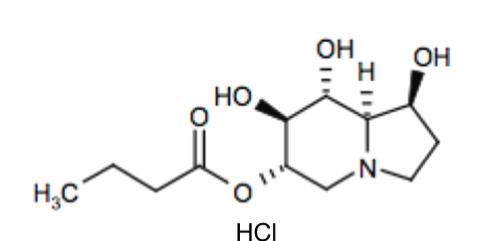


Figure 1: Chemical structure of celgosivir hydrochloride

Celgosivir is supplied as a capsule formulation containing 100 mg drug substance. No excipients are formulated with the drug. The drug is manufactured by Dalton Pharma Services, Inc., Toronto Canada at a GMP facility that holds a current Health Canada Establishment license.

Placebo is supplied as an identical capsule containing 100 mg pregelatinized maize starch. The placebo material conforms to the legal requirements of the US and European Pharmacopoeia.

Capsules should be stored at room temperature (15°C ± 30°C) at relative humidity <65%.

## Dosing Regimen

Patients will be given an initial loading dose of 400 mg (4capsules). Subsequent dosing will be 200 mg (2 capsule) every 12 hr. The patient will receive a total of nine (9) doses, one 400 mg dose and eight (8) 200 mg doses. The capsule(s) should be administered with ~15 mL water. Drug should be taken 1 hr before consuming food or 2 hr afterwards.

## Diet

To mitigate expected gastrointestinal effects (diarrhea and flatulence), patients will be placed on a special diet of protein, vitamins, and glucose containing minimal complex sugars or starches. Patients should be advised to avoid ingestion of sweets or starchy foods while on study drug.

## Concomitant Medications

Patients may continue their baseline medication(s). Patients may be administered paracetamol according to the Principal Investigator’s or Co-investigators’ instructions. The daily dose of all medications should be recorded on the appropriate pages of the CRF.

## Drug Accountability

Upon receipt of drug product, the pharmacist affiliated with the IMU will be responsible for taking an inventory of the investigational drug and placebo agents. A record of this inventory must be kept and usage must be documented on drug inventory forms. The record must be available for inspection by the medical monitor, IRB or regulatory authorities.

# Study Procedures

## Methods and Assessments

Patients who present at the polyclinics, Tan Tock Seng Hospital, at the Emergency Departments (ED), SGH and NUH, at general practitioner’s office, or are self-referred, male or female, age 21 to 60, who have fever ≥ 38°C of ≤ 48 hr duration, at least two criteria indicating probable dengue infection, and a positive NS1 strip assay will be referred to the Investigational Medicine Unit (IMU) to undergo screening. Eligible patients will be approached and invited for participation. The patient will be given the patient information sheet and allowed to ask any questions about the trial. The Principal Investigator or a Co-investigator will take informed consent. Patients meeting all inclusion and exclusion criteria and who agree to participate will be enrolled and admitted to the Investigational Medicine Unit (IMU).

A flowchart showing the patients’ activities throughout the whole study is shown in . A table summarizing the assessment schedule for the study is shown in the Appendix, Table 2.

Upon admission, a clinical examination will be conducted, including ECG and chest X-ray. Blood samples will be taken for baseline assessments - viremia, quantitative NS1, IgG specific for previous dengue infection, hematology and coagulation, clinical chemistry, immunological profile, pre-dose pharmacokinetics, and DNA and RNA analysis. Urine will be collected for a pregnancy test (female patients only) and urine protein/ creatinine ratio and urinalysis (all patients). The patient will be asked to complete a visual analog scale for pain (VASP). The patient will then be randomized to placebo or celgosivir group. Capsules will be administered without food from Day 1 to Day 5. Patients will be placed on a special diet of protein, vitamins, and glucose containing minimal complex sugars or starches. Oral fluid intake, urine output, and stool frequency and consistency will be recorded, as well as any adverse events. Daily clinical exams will be conducted. Blood samples will be drawn daily while in hospital for hematology and clinical chemistry, viremia, quantitative NS1 levels, and immunological profiling (RNA expression levels and cytokines). Any subject who has a platelet count drop of ≥50,000/mm^3^ over a 24 hour period will require full blood count monitoring every 12 hr henceforth until platelet count trend reverses. Urinalysis will be conducted daily, and a sample of urine will be reserved for PK drug levels. Patients also will be asked to complete a VASP daily. Pain medications and any other concomitant medications will be recorded (date/time of ingestion and amount). Over the course of the hospital stay, eight blood samples will be obtained for pharmacokinetic assessments: one pre-dose sample will be taken on Study Day 1, three samples will be obtained on Study Days 2 through 4 between 1 to 3 hr after ingestion of drug (peak levels), and four samples will be obtained on Study Days 2 through 5 immediately prior to ingestion of the next dose (trough levels). The date and time of pharmacokinetic blood draws will be recorded on the CRF. On Study Day 5, after blood draws, administration of the last dose, safety assessments, determination of risk for DHF or DSS, and satisfactory clinical status, the patient will be discharged. Patients at risk for or who have dengue hemorrhagic fever (DHF) or dengue shock syndrome (DSS) will be transferred to a medical ward of the hospital until fit for discharge. Patients who progress to DHF or DSS may require intravenous fluids, platelet and/or blood transfusions and evaluations from other clinical specialists. They will be treated according to standard of care for DHF and DSS--primarily bed rest, fluids, as well as fever and pain medications (supportive therapy).

Patients will be asked to return to the IMU on Study Days 7, 10, and 15 for blood draws, urinalysis, completion of a VASP, and safety assessments. Estimated total blood volume taken per patient is ~ 200 mL during Study Days 1 to 5 and ~ 80 mL for Study Days 7 to 15. The patient will complete the study on Study Day 15 after the investigator has completed the final assessment, unless ongoing adverse events require monitoring. Patients unable to complete the entire course of drug treatment will be asked to complete all planned in-patient and out-patient assessments.

The sponsor will cover all costs for drugs, procedures, consumables and hospitalization stay (Class A ward) related to dengue illness through Day 15 or effects related to celgosivir usage. Participants in the trial will receive compensation for participation in the study.

## Randomization

Enrolled patients will be randomized into one of the two groups in the same ratio (1:1). Randomly permuted blocks will be used to ensure balance over time. The block length will be determined by the randomization statistician and will not be made known to the clinical investigators and site personnel. Randomization will be carried out via a web-based randomization system hosted by the Singapore Clinical Research Institute (SCRI). Only authorized personnel will be allowed to access the system via a password-protected web site available 24 hours a day. Back-up randomization opaque envelopes (and related procedures) will also be prepared and provided to the site in case of any internet failure.

## Blinding

To minimize observer bias, the patients and investigators will be blinded to the treatment assignment to prevent any bias related to the knowledge of the treatment assignment when assessing the outcome. The clinic study team will be blinded to the treatment assignment. The patients will be identified by a study number unrelated to the treatment assignment, and all forms will be standardized regardless of the treatment assignment. If any serious adverse events occur, the Data Safety Monitoring Board will review the data after unblinding.

## Data Safety Monitoring

An independent Data Safety Monitoring Board (DSMB) will be formed to review the safety in this study. The DSMB will be comprised of three members: a clinician, a clinical pharmacologist and a biostatistician, all independent from the trial. One interim analysis of safety will be performed by the DSMB after safety data are available on twelve (12) patients (6 patients on each randomized arm of treatment). The data will be unblinded for the DSMB review. Any dengue death, admission to the intensive care unit or severe dengue (DHF/DSS) will trigger a data safety review by the DSMB. The decision to terminate the trial prematurely will depend on not just the interim results but other relevant information such as the patients' prognosis and the nature of treatment, as well. Emergency code-break envelopes will be prepared by the randomization statistician and be available for DSMB members. The envelopes will be kept unopened unless unblinding is needed.

The following data will be analysed in the safety assessments: clinical exam (body weight, vital signs); fluid administration; urine output and urinalysis; adverse events including stool frequency and consistency. Adverse events and serious adverse events that will be monitored include but are not limited to the following:

(1) Gastrointestinal side effects such as diarrhea and bloatedness/flatulence.

(2) Severe dengue (DHF/DSS).

(3) Admission to intensive care unit.

(4) Death.

All SAEs which fulfill the above and what is stated in the CIRB LSAE reporting form will be reported to Health Science Authority and Singhealth IRB in accordance with established timelines.

## Data Management

Direct data capture of demographic and clinical data will be conducted using computer notebooks. The data will be kept confidential in password protected computers accessible only by selected research staff. Identifiers will be kept in a separate file in another office and every effort will be made to protect the privacy of the participants. The data to be analyzed will contain only de-identified data. An electronic data capture system will be used. Authorized personnel will be assigned user IDs and passwords to gain access to the database. Data will be entered into database directly by site personnel. Entered data will be systematically checked by built-in edit checks. Data that failed the edit checks will be highlighted to the site personnel for resolution. Data will be managed by SCRI. Electronic data capture will be used. Authorized personnel will be assigned user IDs and passwords to gain access to the database. Data will be entered into database directly by site personnel. Entered data will be systematically checked by SCRI data management staff and validated via database edit checks and database listings. Data queries will be channeled to the responsible parties for resolution.

## Criteria for Discontinuation of Patient Study Drug and for Study Termination

A patient’s study drug will be discontinued for

1) any patient developing severe dengue based on WHO criteria or

2) any patient developing grade 3 severity diarrhoea

The study will be terminated at any time that the Data Safety and Monitoring Board members conclude that:

1) Clear and significant harm is noticed in the treatment;

2) ≥30% serious adverse reactions occur in the treatment arm that are possibly, probably or definitely related to study drug;

3) ≥30% patients develop severe dengue based on WHO criteria;

4) ≥30% patients develop grade 3 severity diarrhoea;

5) The drop out rate is too high (>30%).

Figure 2 Flowchart of patients’ activities

Placebo

(N = 25)

Celgosivir

(N = 25)

Drug administration

Drug administration

Daily assessment

Daily assessment

Return to IMU for assessment

Return to IMU for assessment

Visit to polyclinic or GP/ED of NUH or SGH

Assessment of high probability of dengue infection

Fever ≥38° of ≤ 48 hr duration

≥2 symptoms of dengue fever

Positive NS1 strip assay

Others

Referral to IMU for screening of other inclusion & exclusion criteria

Written inform consent

Baseline assessment

Randomization

Discharge

End of Study

Day 5

Day 1

Day 1 to Day 5

Days 7, 10, 15

Day 15

Prior to Day 1

# Statistical Considerations

## Sample size calculation

To achieve 80% power to detect a difference of one log viremia reduction between the celgosivir treated group and the placebo group, assuming a standard deviation of one log, a sample size of 14 for each group (28 total) is required for a 5% one-sided type I error. With a sample size of 25 in each group (50 total), we are able to detect a difference of 0.7 log viremia reduction at a standard deviation of one log (equivalently a one log reduction at a standard deviation of 1.4 log) at 80% power.

## Statistical analysis

A detailed statistical analysis plan will be prepared prior to unblinding.

General

Continuous variables will be summarized using mean and standard deviation. Categorical variables will be summarized by frequency and percentage. Percentages will be rounded to one decimal place and, therefore, may not always add up to 100. All statistical tests will be set at a 5% level of significance. All confidence intervals will be 95% and two-sided. Statistical analyses will be performed using SAS version 9.3.

Demographic and baseline characteristics

Descriptive statistics will be used to summarize demographics and baseline information and to assess balance between the two groups (mean, standard deviation, minimum, maximum, and median for continuous variables; frequency and percent for categorical variables).

Analysis population

Statistical analysis for efficacy data will be carried out on an intention-to-treat (ITT) basis. An ITT set is defined as all patients who signed the informed consent form for the study enrollment and obtained an enrollment number. The treatment group of patients in the ITT set is the planned treatment group, i.e., according to the randomization list planned prior to the study commencement.

Statistical analysis for safety data will be carried out on an as-treated basis. An as-treated set is defined as all subjects who signed the informed consent form for the study enrollment and obtained an enrollment number. The treatment group of patients in the as-treated set is according to the treatment actually received.

Primary outcome

The primary outcomes include the mean of virological log reduction at Day 2, Day 3 and Day 4 and fever AUC above baseline. The virological log reduction of each patient will be measured at Day 2, Day 3 and Day 4 when they are inpatients. The mean of these three measurements will be computed for each patient, and then be compared between the two groups by a two-sample, one-sided t-test. The body temperature of each patient will be measured at baseline and from Day 1 to Day 5, and a curve of the temperature will be plotted. The area under this curve but above the baseline value will be computed for each patient, and then be compared between the two groups by a two-sample, one-sided t-test.

Secondary outcomes

Viral clearance is indicated by an “undetectable” lab test results in the Dengue PCR at baseline, Day 2, Day 3, Day 4, Day 5 and Day 7. The time from baseline to viral clearance will be analyzed using a Kaplan-Meier analysis.

Serum NS 1 is measured by lab test results of “Dengue Non-structure protein 1”. The mean serum NS 1 between day 2 and Day 5 inclusive will be computed for each patient, and compared between the two groups using linear regression, adjusting for primary vs secondary infection. NS1 clearance is indicated by an undetectable lab test result in the NS1 assay. The time to NS1 clearance will be analyzed using a Kaplan-Meier analysis and compared using log rank test.

Leukocyte is measured by lab test results of “Urinalysis”, while platelet count is measure by the lab test results of “Hematology”. Both outcomes will be measured at baseline, Day 2, Day 3, Day 4 and Day 5. The maximum changes from baseline in leukocyte and platelet counts between Day 2 and Day 5 will be computed for each patient, and compared between the two groups using a two-sample t-test.

Hemoconcentration is defined as the hemaocrit at each day compared to that at Day 15. If the reference value at Day 15 is not available, use the value at Day 10. If both values at Day 10 and Day 15 are not available, the patient will be treated as a missing case. The mean maximal hemoconcentration from Day 2 to Day 5 will be computed for each patient, and compared between the two groups using a two-sample t-test.

Clearance celgosivir and volume of distribution of celgosivir will be obtained from concentration-time date by standard PK analysis. The correlation of these PK parameters with weight, age, and sex will be examined by nonlinear mixed effects modeling.

Exploratory outcomes

The various cytokines including TNF-α, IL-6, IL-12, interferon-γ, and MCP-1, are measured by lab test results of “Cytokines” at Study Days 1, 2, 3, 4, 5, 7, 10, and 15. The maximal change from baseline (Day 1) in these cytokines between Day 2 and 15 will be computed for each patient, and compared between the two groups using two-sample t-test.

To investigate whether patients taking celgosivir will use less analgesic medications during the study, analgesic medications recorded during the in-hospital stay and the out-patient diary will between totaled. The total will be compared between the two groups using Fisher exact test.

To investigate whether patients taking celgosivir will experience a lower intensity of joint and muscle pain during the study, the intensity of joint and muscle pain recorded in the “Pain Score” section during the in-hospital stay and the outpatient diary will be obtained and compared between the two groups using two-sample t-test.

Safety outcome

Safety data will be tabulated according to the standard IMU grading scales for occurrence. The proportion of AEs and SAEs among patients will be reported by cohort.

# Safety Assessment

## Definitions

An **adverse event** (AE) is defined as any untoward medical occurrence in a patient administered a medicinal product, which does not necessarily have a causal relationship with this treatment.

A **serious adverse event** (SAE) is defined as any untoward medical occurrence that at any dose results in death, is life-threatening, requires inpatient hospitalization or prolongation of existing hospitalization, results in persistent or significant disability/incapacity or is a congenital anomaly/birth defect.

## Adverse Event Grading, Causality, and Expectedness

The investigator will evaluate all adverse events with regard to severity, the relatedness to the investigational product, and the expectedness. All AEs will be graded based on standard Adverse Event Severity Grading Tables. In the event that an AE does not have an Adverse Event Severity Grading, the following severity classifications will be used:

| **Mild** | causing no limitation of usual activities |
| --- | --- |
| **Moderate** | causing some limitation of usual activities |
| **Severe** | causing inability to carry out usual activities |

The causal relatedness of an AE may be one of the following:

| **Definitely Related** | reasonable temporal relationship to study drug administration  follows a known response pattern (i.e., drug is known to cause this AE)  there is no alternative etiology |
| --- | --- |
| **Probably Related** | reasonable temporal relationship  follows a suspected response pattern (e.g. based on pharmacology)  no evidence for a more likely alternative etiology |
| **Possibly Related** | reasonable temporal relationship  little evidence for a more likely alternative etiology |
| **Probably Not Related** | does not have a reasonable temporal relationship  good evidence for a more likely alternative etiology |
| **Not Related** | does not have a temporal relationship  definitely due to alternative etiology |

The expectedness of an AE may be either expected or unexpected

| **Expected** | Nature and severity of reaction is consistent with information in the Investigator’s Brochure |
| --- | --- |
| **Unexpected** | Nature and severity of reaction is not consistent with information in the Investigator’s Brochure  Specificity or severity of the AE is greater than previously known. |

For AEs, event terminology, the date and time of event start and end, severity, relatedness, impact to the continuation of the study, and final outcome of the event will be recorded on the CRF. For a SAE, in addition to the above, an event summary, the criteria used to categorize the event as an SAE, and a list of all tests and treatments given for the even should be documented.

## Serious Adverse Event Reporting

Serious adverse events require expedited reporting.

Serious adverse events, whether or not they are thought to be related to the investigational drug, must be reported to the study monitor within 24 hours. Within that time, a completed Serious Adverse Event worksheet and other available supporting documentation must be forwarded to the study monitor by facsimile to:

CELADEN Study Monitor

Singapore Clinical Research Institute

Tel No. 6508 8311

Fax: 6508 8317

All patients with serious adverse events must be followed for outcome.

For SAEs, related and unexpected resulting in death or life-threatening events, an initial report must be made to the Singhealth Institutional Review Board and the Clinical Trials Branch of the Health Sciences Authority within 7 calendar days of the event. For SAEs, related and unexpected resulting in non-fatal or non-life threatening events, notification to must occur within 15 calendar days of the event. Refer to the Health Science Authority’s Guidance “Safety Reporting Requirements for Clinical Trial Drugs” for further details of reporting requirements.

# Study Monitoring

The study monitor will visit the IMU routinely throughout the trial. The monitor will review CRFs and compare them with source documents to verify accurate and complete collection of data and confirm that the study is being conducted according to the protocol, Good Clinical Practices (GCP) and all applicable regulations.

# References

Courageot MP, Frenkiel MP, Dos Santos CD *et al.* (2000). "Alpha-glucosidase inhibitors reduce dengue virus production by affecting the initial steps of virion morphogenesis in the endoplasmic reticulum." *Journal of Virology* **74**(1): 564-72

Durantel D (2009). "Celgosivir, an alpha-glucosidase I inhibitor for the potential treatment of HCV infection." *Curr Opin Investig Drugs* **10**(8): 860-70

Fink J, Gu F and Vasudevan SG (2006). "Role of T cells, cytokines and antibody in dengue fever and dengue haemorrhagic fever." *Rev Med Virol* **16**(4): 263-75

Halstead SB (2007). "Dengue." *Lancet* **370**(9599): 1644-52

Kaita K, Yoshida E, Kunimoto D *et al.* (2007). "Phase II Proof of Concept Study of Celgosivir in Combination with Peginterferon alfa-2b and Ribavirin in Chronic Hepatitis C Genotype-1 Non Responder Patients." *Digestive Disease Weekly* **May**: Abstr 442

Rathore APS, Paradkar PN, Watanabe S *et al.* (2011). "Celgosivir treatment misfolds dengue virus NS1 protein, induces cellular pro-survival genes and protects against lethal challenge mouse model." *ANTIVIRAL RESEARCH* **92**(3): 453-60

Remme JH, Blas E, Chitsulo L *et al.* (2002). "Strategic emphases for tropical diseases research: a TDR perspective." *Trends Parasitol* **18**(10): 421-6

Schul W, Liu W, Xu H-Y *et al.* (2007). "A dengue fever viremia model in mice shows reduction in viral replication and suppression of the inflammatory response after treatment with antiviral drugs." *J INFECT DIS* **195**(5): 665-74

Sorbera LA, Castaner J and Garcia-Capdevila L (2005). "Celgosivir." *Drugs of the Future* **30**(6): 545-552

Stoltz M, McPherson M, Frampton M *et al.* (1996). "PK of celgosivir in asymptomatic HIV-positive patients treated with MDL 28,574A during phase I trials." *Int Conf AIDS Abstracts*: 1-1

WHO (2009). "Dengue and dengue hemorrhagic fever fact sheet 117." *World Health Organization*

Table 2 Timeline of study assessments

|  | Screen-ing | Baseline (Prior to 1st dose) | Dosing and Monitoring Phase | | | | | | | | | | | | | | | | | Monitoring Phase | | | |
| --- | --- | --- | --- | --- | --- | --- | --- | --- | --- | --- | --- | --- | --- | --- | --- | --- | --- | --- | --- | --- | --- | --- | --- |
| Hospital Status |  | In-patient | | | | | | | | | | | | | | | | | | | Out-patient | | |
| Study Day | 1 | | | | | 2 | | | | 3 | | | | 4 | | | | 5 | | | 7 | 10 | 15 |
| Time (Hr) relative to first dose | -14 to 0 | | **0+/-1hr** | 0 to 3 | **11 to 12+/-1hr** | 23 to 24 | **24+/-1hr** | 24 to 27 | **35 to 36+/-1hr** | 47 to 48 | **48+/-1hr** | 48 to 51 | **59 to 60+/-1hr** | 71 to 72 | **72+/-1hr** | 72 to 75 | **83 to 84+/-1hr** | 95 to 96 | **96+/-1hr** | 96 to 99 | 144 ± 24 | 216 ± 24 | 336 ± 24 |
| **Evaluation & Administrative Assessments** |  | |  |  |  |  |  |  |  |  |  |  |  |  |  |  |  |  |  |  |  |  |  |
| Demographics | + | |  |  |  |  |  |  |  |  |  |  |  |  |  |  |  |  |  |  |  |  |  |
| Medical History | + | |  |  |  |  |  |  |  |  |  |  |  |  |  |  |  |  |  |  |  |  |  |
| Informed Consent | + | |  |  |  |  |  |  |  |  |  |  |  |  |  |  |  |  |  |  |  |  |  |
| NS1 Strip Assay - test kits provided by Duke | + | |  |  |  |  |  |  |  |  |  |  |  |  |  |  |  |  |  |  |  |  |  |
| Clinical Exam ^h^ | + | |  |  | ~~+~~ | ~~+~~ |  |  | ~~+~~ | ~~+~~ |  |  | ~~+~~ | ~~+~~ |  |  | ~~+~~ | ~~+~~ |  | ~~-~~ | ~~+~~ | ~~+~~ | ~~+~~ |
| Body Weight and Height ^a^ | + | |  |  | + | + |  |  | + | + |  |  | + | + |  |  | + | + |  |  | + | + | + |
| Vital Signs (Body T^b^, BP ^c^, PR ^c^, O_2_ saturation ^c^) | + | |  | + | + | + |  | + | + | + |  | + | + | + |  | + | + | + |  | + | + | + | + |
| Hematology (FBC and WBC differential) - EDTA tube^g^ | + | |  |  |  | + |  |  |  | + |  |  |  | + |  |  |  | + |  |  | + | + | + |
| PT/PTT | + | |  |  |  |  |  |  |  |  |  |  |  |  |  |  |  |  |  |  |  |  |  |
| Clinical chemistry (full renal and liver panel) - plain tube | + | |  |  |  | + |  |  |  | + |  |  |  | + |  |  |  | + |  |  | + | + | + |
| GXM (PRN)^d^ |  | |  |  |  |  |  |  |  |  |  |  |  |  |  |  |  |  |  |  |  |  |  |
| ECG | + | |  | + |  |  |  | + |  |  |  | + |  |  |  | + |  |  |  | + | + |  | + |
| Chest X-ray | + | |  |  |  |  |  |  |  |  |  |  |  |  |  | + |  |  |  |  |  |  |  |
| Pregnancy Assay in Females (Urine) | + | |  |  |  |  |  |  |  |  |  |  |  |  |  |  |  |  |  |  |  |  |  |
| Urinalysis | + | |  |  |  | + |  |  |  | + |  |  |  | + |  |  |  | + |  |  | + |  |  |
| Urine protein/creatinine ratio | + | |  |  |  | + |  |  |  | + |  |  |  | + |  |  |  | + |  |  | + |  |  |
| Dengue IgM |  | |  |  |  |  |  |  |  |  |  |  |  |  |  |  |  |  |  |  | + |  |  |
| Dengue IgG | +  + | |  |  |  |  |  |  |  |  |  |  |  |  |  |  |  |  |  |  |  |  |  |
| Drug Administration (capsules) |  | | 4 |  | 2 |  | 2 |  | 2 |  | 2 |  | 2 |  | 2 |  | 2 |  | 2 |  |  |  |  |
| Viremia | + | |  |  |  | + |  |  |  | + |  |  |  | + |  |  |  | + |  |  | + |  |  |
| Quantitative NS1 | + | |  |  |  | + |  |  |  | + |  |  |  | + |  |  |  | + |  |  | + | + | + |
| Immunological Profile (Cytokines) | + | |  |  |  | + |  |  |  | + |  |  |  | + |  |  |  | + |  |  | + | + | + |
| ~~DNA~~ | ~~+~~ | |  |  |  |  |  |  |  |  |  |  |  |  |  |  |  |  |  |  |  |  |  |
| RNA | + | |  |  |  | + |  |  |  | + |  |  |  | + |  |  |  | + |  |  | + | + | + |
| PK samples - serum ^e^ | + | |  |  |  | + |  | + |  | + |  | + |  | + |  | + |  | + |  |  |  |  |  |
| PK sample - urine ^f^ | + | |  |  | + | + | + |  | + | + |  |  | + | + |  |  | + | + |  |  |  |  |  |
| Visual Analog Scale for Pain | + | |  |  |  |  |  | + |  |  |  | + |  |  |  | + |  |  |  | + | + | + | + |
| Record Fluid Administration |  | |  | + |  |  |  | + |  |  |  | + |  | + |  | + |  |  |  | + |  |  |  |
| Verify Urine production (I/O)^c^ |  | |  | + |  |  |  | + |  |  |  | + |  | + |  | + |  |  |  | + |  |  |  |
| Record Adverse Events |  | |  | + |  |  |  | + |  |  |  | + |  |  |  | + |  |  |  | + | + | + | + |
| Record Stool Freq & Consistency & Flatulence |  | |  | + |  |  |  | + |  |  |  | + |  |  |  | + |  |  |  | + | + | + | + |
| Record Pain Medications | + | |  | + |  |  |  | + |  |  |  | + |  |  |  | + |  |  |  | + | + | + | + |
| Record Other Concomitant Meds | + | |  | + |  |  |  | + |  |  |  | + |  |  |  | + |  |  |  | + | + | + | + |

Annotations:

^a^ Height to be performed at screening visit only

^b^ 2 hourly temperature monitoring

^c^6 hourly BP, PR, O2 saturation and I/O monitoring

^d^ Group and Cross March (GXM) to be perform if clinical indication by study investigator only

^e^ PK samples – serum perform at (Pre-dose, 23hr(+/-1hr) , 25 hr(+/-0.5hr), 47hr(+/-1hr), 49.5hr(+/-0.5hr), 71hr(+/-1hr), 74hr(+/-1hr)and 95hr(+/-1hr))

^f^ PK sample – urine perform at (Pre-dose, 0 – 12hr, 12hr – 24hr, 24h – 36hr, 36hr – 48hr, 48hr – 60hr, 60hr – 72hr, 72hr – 84hr, 84hr – 96hr)

^g^ Any subject who has a platelet count drop of ≥50,000/mm^3^ over a 24 hour period will require FBC monitoring every 12 hr henceforth until platelet count trend reverses.

^h^ Clinical exam will be performed twice a day (except for Study Day 5).

| Send collected samples to Duke-NUS |
| --- |
| Send PK samples to Prof HS Lee @ NUS |
